# Identifying variation in dinosaur footprints and classifying problematic specimens via unbiased unsupervised machine learning

**DOI:** 10.1073/pnas.2527222122

**Published:** 2026-01-26

**Authors:** Gregor Hartmann, Tone Blakesley, Paige E. dePolo, Stephen L. Brusatte

**Affiliations:** ^a^Department of Optics and Beamlines, Helmholtz-Zentrum Berlin für Materialien und Energie GmbH, Berlin 12489, Germany; ^b^School of GeoSciences, University of Edinburgh, Edinburgh EH9 3FE, Scotland, United Kingdom; ^c^School of Biological and Environmental Sciences, Liverpool John Moores University, Liverpool L3 3AF, England, United Kingdom

**Keywords:** dinosaurs, footprints, trace fossils, AI, machine learning

## Abstract

Machine learning holds great promise for classifying and identifying fossils, and has recently been marshaled to identify trackmakers of dinosaur footprints and address long-standing debates over whether some dinosaur tracks are the oldest birds or ornithopods (duck-billed herbivores and kin) in the fossil record, or alternatively were made by nonavian theropods. Existing methods in paleontology, however, require supervision and a priori labeling of training data by researchers, which can lead to bias. We employ an unsupervised machine learning technique for recognizing inherent patterns in shape data, using a disentangled variational autoencoder network, to a database of 1,974 footprints, spanning a diversity of dinosaurs across their evolutionary history, including modern birds. Our neural network identified eight features of shape variation that most differentiate these tracks: overall load and shape (amount of ground contact area), digit spread, digit attachment, heel load, digit and heel emphasis, loading position, heel position, and left–right load. With the unsupervised process finished, we a posteriori labeled each track based on published expert judgments, plotted them into morphospace, and applied distance metrics to group means and nearest neighbors, which showed 80 to 93% agreement with expert identifications. Controversial Late Triassic-Early Jurassic bird-like tracks group with fossil and modern birds and some Middle Jurassic three-toed tracks with ornithopods, supporting an older origin for these groups than recorded by body fossils. We provide an app, DinoTracker, to make this process accessible, and source code that can be adapted to other cases where paleontologists or biologists are studying patterns of shape variation.

## Significance

Dinosaur footprints are iconic fossils, but it is challenging to identify their makers. This is illustrated by a long-standing debate about whether some footprints from the Late Triassic-Early Jurassic belong to birds, which would be ~60 Ma older than the oldest skeletons. Recently, machine learning has been heralded as a tool for classifying and identifying tracks, but existing methods require researchers to supervise the process by labeling training data, which can perpetuate human biases. We use an unsupervised neural network to process a dataset of nearly 2,000 dinosaur tracks, which recognizes eight ways in which they most vary, and which finds that the problematic bird-like tracks are more similar to modern and fossil birds than any other dinosaur.

In recent years, machine learning has been touted as a new frontier in classifying and identifying fossils (1–3). Some of the first heralded case studies endeavored to identify trackmakers of dinosaur footprints (4–7). This is an important goal, as footprints and other trace fossils such as burrows, which record the activities of ancient organisms, are often more numerous than bones, shells, and other physical remains of the organisms themselves (body fossils) (8, 9). Because trace fossils reflect interactions between animals and their environments, they can reveal aspects of behavior difficult to discern from body fossils, such as preferred habitats and locomotion styles (10–12). Furthermore, because trace fossils can be locally abundant and are embedded into the sediments in which they formed and cannot be transported like a body fossil, they provide critical information on the distribution and origination times of species and groups. Indeed, major groups such as tetrapods (limbed land-living vertebrates), amniotes (fully terrestrial tetrapods), and dinosauromorphs (dinosaurs and their closest kin) are purported to appear in the fossil record first as footprints, sometimes many millions of years before their oldest body fossils (13–15).

It is difficult, however, to determine which features are most useful in distinguishing footprints from each other and in identifying their makers. Unless a skeleton is fossilized in its tracks, a footprint cannot be matched definitively with its creator. Sometimes unique features of a trackmaker’s foot can register in a footprint and help recognize a trackmaker, but this “synapomorphy-based” method (16, 17) is limited because not all dinosaurs have bespoke attributes of their feet, and even if they do, they might be subtle and not easily preserved in footprints. Additionally, track shape is influenced by dynamic interaction of factors beyond the anatomy of the foot, including limb motion and substrate properties (18). Therefore, most dinosaur tracks are classified and assigned to general trackmaker groups based on their overall size and shape, as determined qualitatively or semiquantitatively by experts. Occasionally this is straightforward and results in widespread agreement among experts, as with bathtub-sized footprints that could only be made by colossal sauropods (17). Often, however, it is challenging, as illustrated by long-standing debates whether certain three-toed tracks were made by herbivorous ornithopods or carnivorous theropods (4, 19–21), and whether various bird-like tracks were made by true birds or closely related theropods (22–24). The latter conundrum, in particular, has broad implications for understanding the timing of avian origins, and the environments in which birds evolved flight.

Machine learning has great potential to recognize patterns in footprint shape and find matches with trackmakers. Recent studies have delivered promising results (4–7), and demonstrate the potential for machine learning to contribute to paleontology with its ability to notice patterns, untangle variation, and classify data. Yet, previous studies have one key limitation: their computational neural networks need to be supervised. This is true of nearly every paleontological application of machine learning thus far (1, but see ref. 25). For instance, in their pioneering study inaugurating the use of machine learning in identifying dinosaur tracks, Lallensack et al. (4) had to first train their neural network with a training dataset of tracks labeled as either theropod or ornithischian (~ornithopod), as determined a priori by human observers. If these identifications are wrong, then the output of the machine learning model will be biased. Unsupervised methods that circumvent this limitation are held as the future of machine learning in paleontology, but are computationally challenging to implement (1).

Here we provide a robust unsupervised method for recognizing inherent patterns in shape data of paleontological specimens, utilizing a disentangled variational autoencoder (β-VAE), and apply it to a case study of dinosaur footprint shape. Unlike previous studies, our approach does not require a priori labeling of tracks (e.g., theropod vs. ornithopod) in a training phase, but rather the neural network algorithm searches in an unsupervised manner for those features that vary independently and that most differentiate the suite of tracks. Our focus, therefore, is fundamentally on characterization of tracks based on their maximum degrees of shape variation. The neural network identifies the most meaningful features of the footprints, and then with this information in hand, the human observer can interpret those features to present informed hypotheses for trackmaker identifications. We make this process user-friendly by providing an app, called DinoTracker (26), which can be downloaded to a mobile or desktop device, into which users can import a silhouette of any track—such as one recently collected in the field—and compare it with the footprints in our dataset and the results of our model. We also provide the source code for our network, which can be adapted to other cases where patterns in fossil or biological shape are being studied.

## Results

**Fig. 1.**
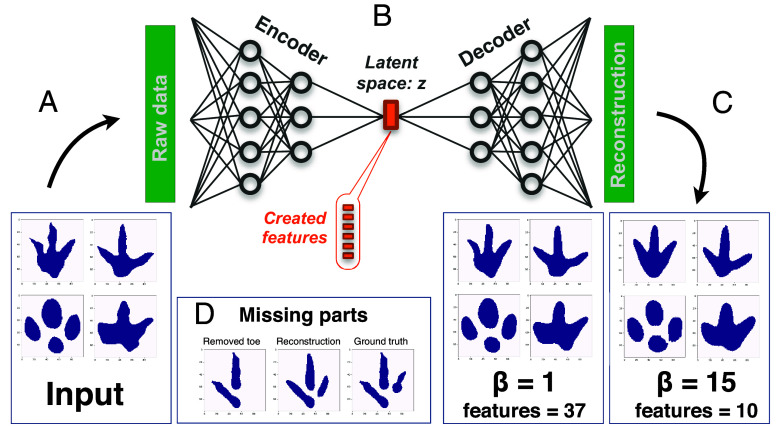
The disentangled variational autoencoder method. Silhouettes of dinosaur footprints (*A*) are processed through an artificial neural network (*B*) with a dimensional bottleneck in its center. Due to a disentanglement condition, whose strength is given by the β parameter, the compressed representation, created by the encoder part of the network and called the latent space z, is interpretable to the human mind. From this state, the decoder reconstructs the data (*C*). The reconstruction is shown for two different β values resulting in a different reconstruction quality and number of features. The method is capable of sensible reconstruction of missing parts (*D*).

The analytical pipeline for our disentangled variational autoencoder method is shown in Fig. 1. It uses an artificial neural network to process our dataset of 1,974 two-dimensional dinosaur footprint silhouettes, which are presented to the network without any taxonomic labels such as “theropod” or “ornithopod.” Details of the network analysis are given in Materials and Methods and *SI Appendix*. During the encoding phase (Fig. 1*A*), the network processes the dataset through a dimensional bottleneck, creating a compressed disentangled representation of the entire dataset (Fig. 1*B*), which is philosophically akin to a multivariate method like principal component analysis (PCA). This creates a latent space that is interpretable for the human mind, unlike the strictly linear PCA that reduces datasets to principal components that are statistical abstractions and thus not necessarily able to be visualized by humans. Then, during the decoding phase (Fig. 1*C*), the network reconstructs the data.

Critical to the creation of the latent space is the β parameter, which determines the strength of the data disentanglement during encoding. β must be optimized, and is a tradeoff between accuracy of the resulting footprint reconstructions in the decoding phase and the number of parameters that the network identifies as key features of footprint variation. The lower the β value, the more accurate the final decoded footprint reconstructions, but the more parameters needed. Our sensitivity analyses show that β = 15 delivers accurate footprints with a low number of used features in contrast to, for instance, β = 1 (Fig. 1*C*), and thus we chose to proceed with β = 15.

As an additional sensitivity analysis and to increase the training data size, we employed data augmentation techniques to test how well the model can reconstruct portions of known footprints if those portions are altered. We modified the footprint silhouettes so they were randomly rotated, mirrored, stretched, or compressed, or to remove part of the track (*SI Appendix*, Fig. S1), as well as slight edge modifications. When the network reconstructed those modified or deleted parts, the reconstructions visually match closely to the original unmodified versions (Fig. 1*D*), lending confidence to the decoding process.

We also analyzed the performance of the network by reserving 4% of the original data (test data) from our 1,974-footprint dataset that was not used in the original model training (train data). When confronted with test data the network is adept at recognizing and classifying variation in tracks that it has yet to see in the training data (*SI Appendix*, Fig. S2), demonstrating that network performance is robust and does not simply memorize.

**Fig. 2.**
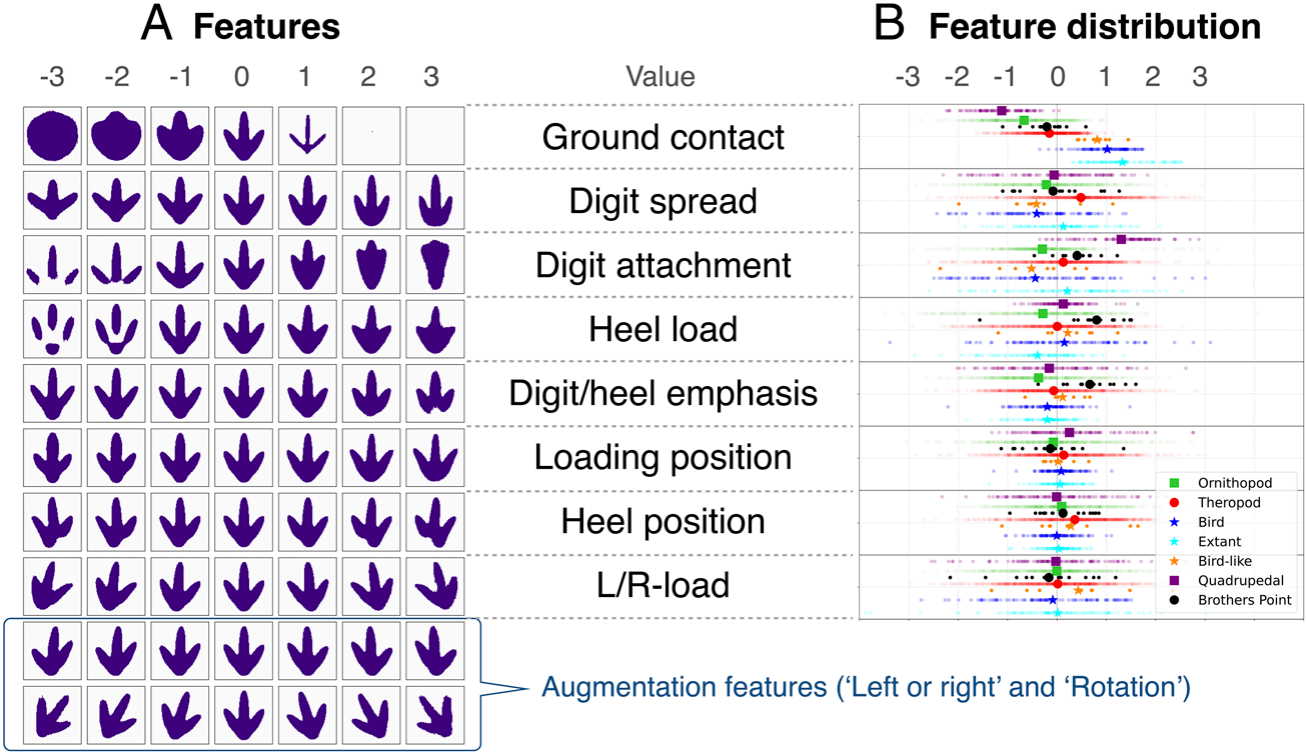
Analysis of the latent space: Ten features are created by the network; these represent the features of maximum independent variation of the tracks. After removal of two related to mirroring and rotation in augmentation, this leaves eight key features in which the tracks vary. (*A*) The influence of each individual feature is shown by varying the feature’s value in the range of [−3,3] while all other features remain unchanged, showing the influence of the respective feature on the reconstruction for the average track of the entire dataset, which is the track shown in the central column. (*B*) The average value and distribution of each key feature are shown for theropods, ornithopods, fossil birds, extant birds, quadrupedal dinosaurs, problematic bird-like tracks, and problematic tracks from Brothers’ Point, Scotland, that may belong to nonavian theropods or ornithopods.

With the robustness of our approach established, we then ran the model (with β = 15). From a maximum number of 50 possible “representations” (limited by the network architecture)—the features of variation among the footprints—the network generated 10 disentangled features which are highlighted in Fig. 2. Among these, two are augmentation features representing rotations and mirroring effects and were excluded (*SI Appendix*) from further analysis. The remaining eight are the features of maximum shape variation in the footprints, and thus the most useful ways to distinguish them. Based on a visual examination of their observed shape range among the tracks (Fig. 2*A*), we interpret them as representing: overall load and shape (amount of ground contact area), digit spread, digit attachment, heel load, digit and heel emphasis, loading position, heel position, and left–right load.

With these features of maximum variation identified, we can interrogate how they relate to trackmaker identifications, by a posteriori labeling each track based on expert identifications in the literature. We classified the tracks into seven groups. Five of these are affirmatively identified by experts: ornithopods, nonavian theropods, fossil birds, extant birds, quadrupedal dinosaurs (including sauropods, stegosaurs, and ankylosaurs). The remaining two are equivocal: tracks that are bird-like but might belong to nonavian theropods (22–24) and tracks from the Middle Jurassic of Scotland that could be either theropod or ornithopod (27, 28). Average values and total distributions for each of the eight footprint features in these seven groups are shown in Fig. 2*B*.

Feature 1, overall load and shape of the track, exhibits the maximum amount of variation, and thus is most important in distinguishing and classifying footprints. Tracks attributed to quadrupedal dinosaurs, ornithopods, nonavian theropods, fossil birds, and extant birds are clearly differentiated in average values, and the total spread of their values (ranges) is staggered relative to each other (Fig. 2*A*1), meaning they map to distinct regions of trait space. Interestingly, tracks attributed to ornithopods and nonavian theropods are closer to each other in this feature than nonavian theropods are to their evolutionary descendants, fossil, and extant birds. The taxonomic groups are less consistently distinguished from each other for the other seven features, although individual traits are often useful in discriminating between certain of the groups—for instance, quadrupedal dinosaurs are highly different from ornithopods and theropods in Feature 3, digit attachment (Fig. 2*A*3).

**Fig. 3.**
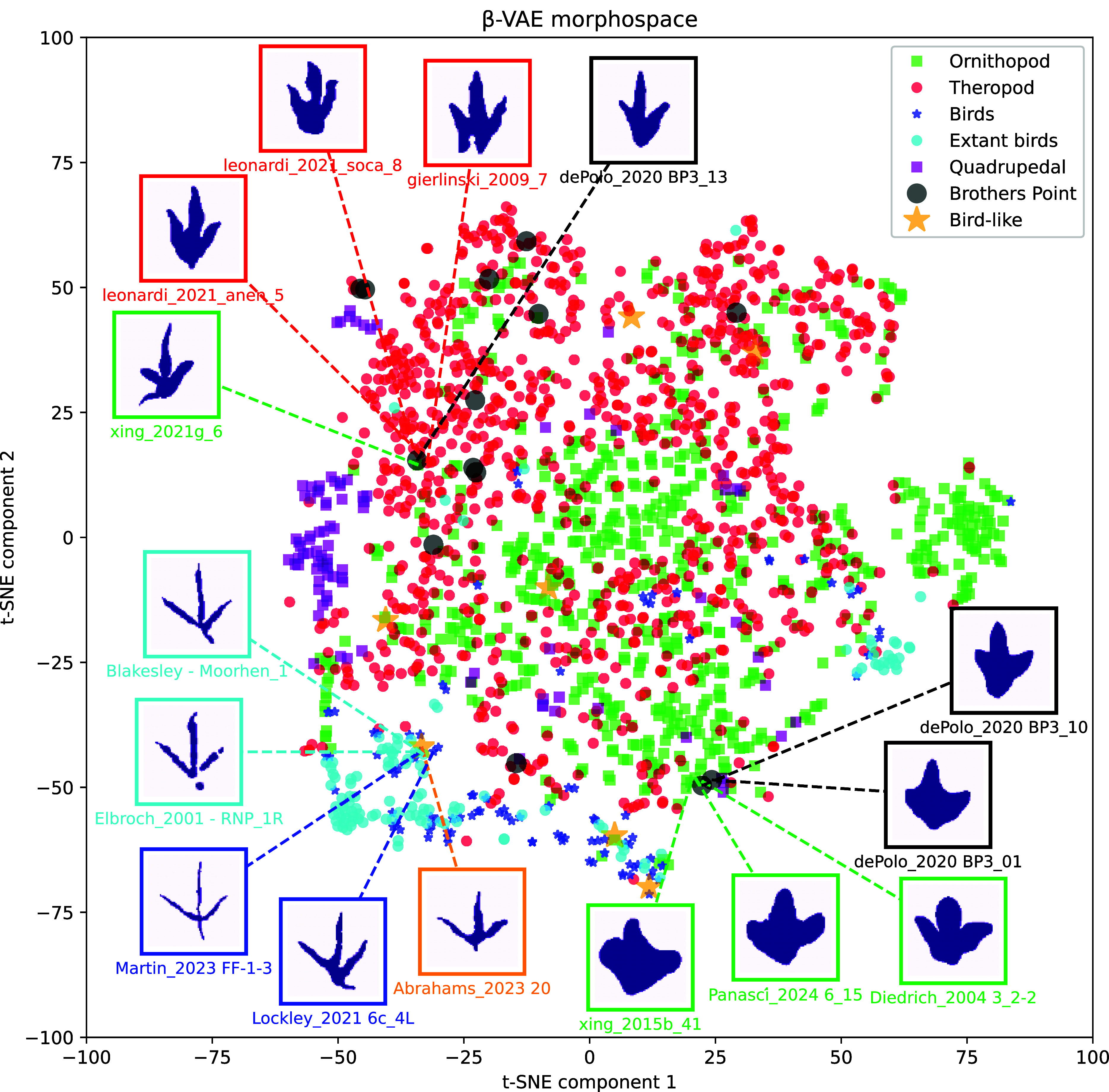
β-VAE morphospace showing the overall spread of variation in the footprints in our database, each assigned to a group a posteriori after the neural network analysis based on expert identification in the literature. The t-SNE algorithm was used to condense the eight-dimensional space (for the eight key footprint features) to a 2D map. Positions of problematic Brothers’ Point (theropod-or-ornithopod) and bird-like tracks are indicated, showing which definitively identified tracks they most closely correspond to. Examples of individual footprints belonging to groups are shown as silhouettes, color-coded to group identity.

To encapsulate and visualize the range of variation among trackmaker groups, and to aid identification of problematic tracks, we distilled information from all eight features into a single two-dimensional β-VAE morphospace map, using t-distributed stochastic neighbor embedding (t-SNE) (Fig. 3). This plot shows broad overlap in places between nonavian theropods and ornithopods, but generally ornithopods have more negative values and nonavian theropods more positive values on the Y axis (second t-SNE component), which helps to distinguish end-members of each group. Quadrupedal dinosaurs form their own cluster at the upper left, and interestingly there are two distinct clusters that both mix modern and extinct birds. Some, but not all, problematic Middle Jurassic tracks cluster more tightly with theropods than ornithopods, and nearly all problematic bird-like tracks group more closely with birds than nonavian theropods.

**Fig. 4.**
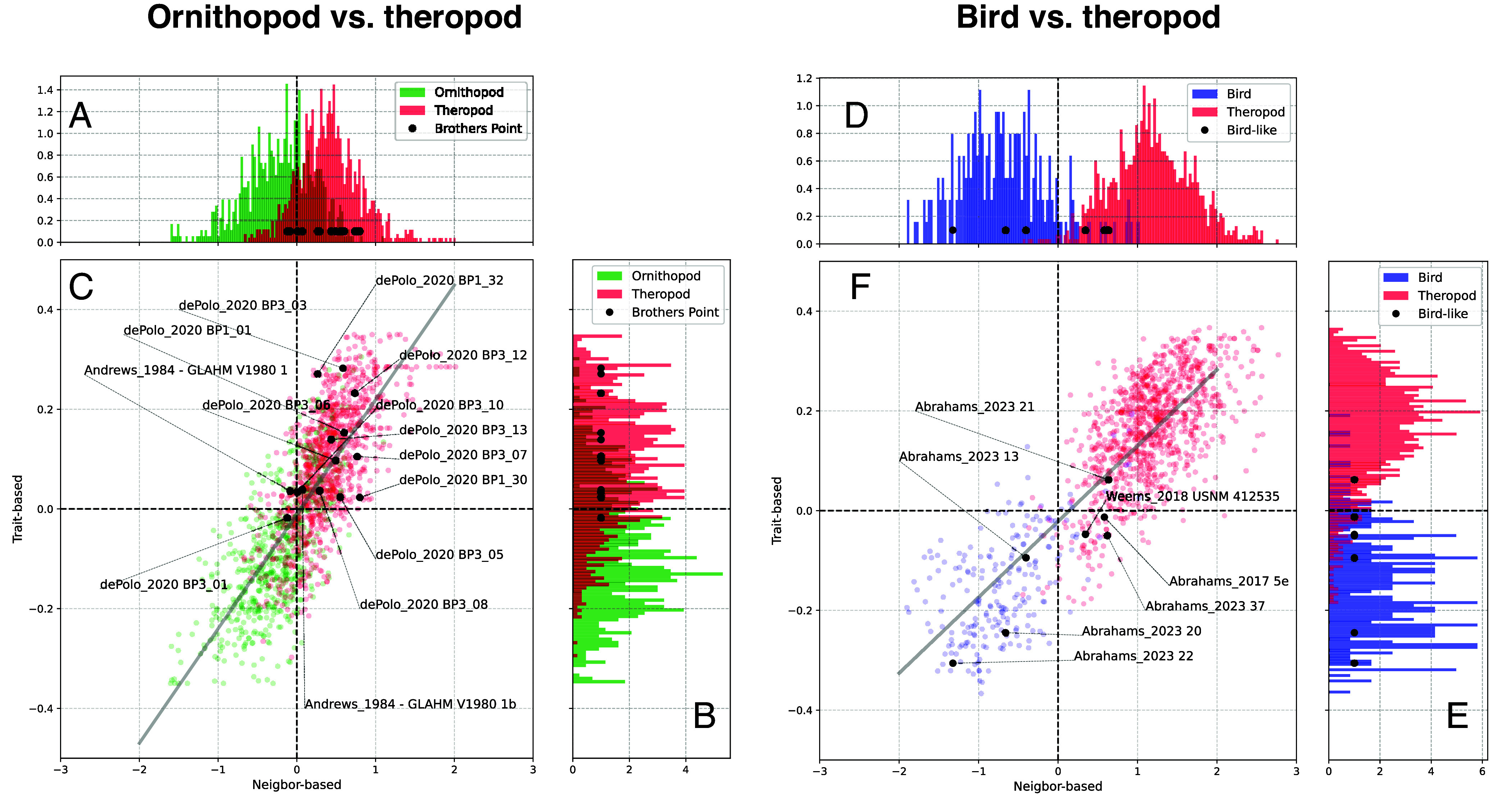
Identifying problematic footprints with distance metrics. (*A*) Histogram of nearest-neighbor distances for nonavian theropods vs. ornithopods. (*B*) Histogram of trait-based distances for nonavian theropods vs. ornithopods. (*C*) Two-dimensional plot comparing the nearest-neighbor and trait-based distances for nonavian theropods, ornithopods, and problematic tracks. (*D*) Histogram of nearest-neighbor distances for nonavian theropods vs. birds. (*E*) Histogram of trait-based distances for nonavian theropods vs. birds. (*F*) Two-dimensional plot comparing the nearest-neighbor and trait-based distances for nonavian theropods, birds, and problematic tracks. In the quadrant maps (*C*, *F*), tracks in the upper left quadrant are identified as nonavian theropods by the trait-based metric and as ornithopods or birds by the nearest-neighbor metric. Tracks in the upper right quadrant are identified as nonavian theropods by both metrics. Tracks in the lower left quadrant are identified as ornithopods or birds by both metrics. Tracks in the lower right quadrant are identified as ornithopods or birds by the trait-based metric and as nonavian theropods by the nearest-neighbor metric. Positions of problematic Brothers’ Point (theropod–ornithopod) tracks and bird-like tracks are shown as black dots, indicating the quadrants to which they are most similar. Most Brothers’ Point tracks fall in theropod-like quadrants, although some fall within the ornithopod range. Most bird-like tracks fall within the bird-dominated regions, and three lie squarely within the bird quadrant.

To more confidently hypothesize trackmaker identifications, we constructed eight-dimensional morphospaces using the eight features of maximum variation identified by the network (Fig. 4), and used distance metrics to compare each track—both those affirmatively identified in the literature and those that are problematic—to potential groups. When nonavian theropods are compared to ornithopods, approximately 80% of affirmative tracks fall within their expected group in the histograms, and the problematic Middle Jurassic tracks are mostly placed within the theropod side of the histograms but some overlap with the ornithopod range. When nonavian theropods are compared to birds, more than 93% of literature identifications are corroborated, and most of the bird-like tracks are placed within the bird range.

## Discussion

Our disentangled variational autoencoder method demonstrates that unsupervised machine learning can be applied to paleontological data to generate meaningful results and address long-standing problems in fossil classification and identification. Our method produces sensible output, in terms of the quality of reconstructed dinosaur footprints when portions are removed, and more importantly, in distilling the complexities of track shape among ~2,000 samples into a set of key features that explain maximum variation and have anatomical and biological meaning. Importantly, our network identifies a particular feature (Feature 1, overall load and shape of the track) that differentiates footprints that experts have classified into nonavian theropod, fossil bird, extant bird, ornithopod, and quadrupedal dinosaur categories. Thus, classes that have been conceived by the human mind are also evident when the footprints are mathematically analyzed without providing information about those classes to the neural network. This speaks to the power of machine learning methods that do not require a priori assumptions in a training phase, but can confront a wealth of paleontological data with an unbiased view and sort through it unsupervised. It also reassures that such methods are not computational “black boxes,” but enumerate patterns that humans have noticed.

Our neural network summarizes the most important variation among a range of dinosaur tracks, spanning bipeds and quadrupeds, titanic giants to tiny species, runners and walkers to fliers, over more than 200 Ma of history from Triassic origins to the birds of today. We identify the overall load and shape of the track—in other words, the amount of ground contact area—as the principal feature of variation among all dinosaurs. Furthermore, aspects of heel load and digit spread, emphasis, and attachment are also important differences. Untangling these features reveals why it has traditionally been difficult to distinguish many nonavian theropod and ornithopod tracks (19–21): they have similar averages and ranges of variation even in overall load and shape—the feature of maximum disparity among dinosaurs—such that nonavian theropods are closer to ornithopods than even their own descendants, birds. Nonavian theropods and ornithopods are also extremely similar in the seven other key traits, and it is only minor differences in average load and digit spread and attachment that can best differentiate them.

Yet, nontheropod and ornithopod tracks can usually be distinguished from each other, when all eight key features are pooled together as in our two-dimensional morphospace and when distance metrics are used to cross-validate track identifications in the literature. Although there is overlap between their ranges in morphospace, around 80% of the time the distance metrics agree with human expert evaluations of individual tracks as either nonavian theropod or ornithopod. This does mean, however, that some tracks are genuinely difficult to classify into either group. The debated footprints that we focus on, from the Middle Jurassic of Scotland (28), mainly fall within the theropod part of morphospace and the distance histograms, but they are in close proximity to, and overlap with, some ornithopod tracks. We conclude that there is more evidence for theropod affinities for most of these tracks, but some are more liable to be ornithopods, which would make them among the oldest members of the group in the global fossil record.

There is considerable debate as to whether some small, three-toed footprints from the Triassic and Early Jurassic were made by birds, as their slender and highly divergent toe impressions are similar to modern bird tracks (22–24). If so, they would predate the oldest bird body fossils by ~60 Ma, necessitating radical revision of the timing and mode of avian origins. When we analyzed seven of these tracks with our neural network, most fell within the bird-dominated region of morphospace and the distance histograms, distinct from nonavian theropods. This included the oldest of these disputed tracks, from the Triassic of South Africa (23). The machine learning analysis, therefore, corroborates patterns noticed by researchers: these tracks greatly resemble birds. One possibility is that these tracks belonged to true birds, which originated much earlier than thought, before the Triassic-Jurassic extinction, but whose body fossils have yet to be found or were not preserved, perhaps because these birds were rare or lived in specific environments not amenable to bone preservation. Alternatively, as widely hypothesized, these tracks may have been made by small Triassic-Jurassic nonavian dinosaurs with feet and/or ground locomotion styles highly convergent on those of birds (i.e., widely divaricated gracile digits) (23, 29), or which made bird-like tracks with traditional nonavian theropod feet simply by chance, because of substrate properties. For example, digits may appear increasingly gracile in wet substrates, leading to the formation of penetrative tracks, or as widely divaricated as bird tracks due to increased mediolateral stability (29, 30). Regardless, body fossils will be required to solve this riddle.

Among the benefits of our approach is that it is explicit and user-friendly. The neural network identifies key features of track shape variation that are understandable to the human eye and can be interpreted to have anatomical or biological meaning. To provide easy access to the network and the analysis pipeline for scientists without machine learning or coding expertise, we provide an app, called DinoTracker, which is described in detail in *SI Appendix*. It allows the users to import their own track silhouette, obtain its encoded features and decoded reconstruction, manipulate the values of the eight key features to gauge their relation to footprint shape, and identify and display tracks in our database that are most similar.

Our study should not be viewed as the development of an all-knowing oracle but rather a type of investigator with a different perspective—an unbiased expert brought to the table. Our specific protocol is a starting point, as it analyzes two-dimensional footprint silhouettes only, and these must be drawn by the user, which could contribute error. We must also remember that substrate variations, trackmaker locomotion, and preservational artifacts can influence the shapes of footprints as preserved, and this will be reflected in the drawn silhouettes (29–31). Therefore, users should be aware that, when inputting silhouettes of unusual tracks such as swim traces and those with extradigital features (e.g., webbing, long metatarsal impressions), our network will only reconstruct the eight features (Fig. 2*A*) present in these tracks rather than whole track morphology. Future implementations can build on our approach, automate the outlining of the tracks, and take into account more information for each footprint: three-dimensional shape and depth, size, substrate type, and age. And regardless, human experts will still be needed to not only provide the source data, but interpret the output of the analysis, as we have done here by interpreting the biological meaning of the eight key track features and comparing the model results to footprint identifications in the literature.

Overall, however, our unsupervised machine learning approach is less biased than a human observer in recognizing variation, and has potential to expand into other areas of paleontology where data need to be sorted, variation catalogued, and classification conflicts settled—such as in phylogenetics and study of macroevolutionary trends over time.

## Materials and Methods

### Dinosaur Track Dataset.

We compiled a dataset of 1,974 dinosaur tracks from the literature and our own fieldwork. We began with the published dataset of Lallensack et al. (4 and references therein), which includes 961 tracks identified as nonavian theropod and 616 identified as ornithopod. To this, we added 117 tracks of extant birds from various literature sources and field observations; 97 tracks of quadrupedal dinosaurs sourced from the literature (including ref. 4), including those attributed to sauropods, stegosaurs, and ankylosaurs; seven images of controversial “bird-like” tracks from the literature (22–24); and 13 Middle Jurassic tridactyl tracks our team has studied on the Isle of Skye, Scotland, which are debated to belong to either theropods or ornithopods (27, 28). For one of these Scottish tracks, we produced two versions to consider different track margins that may help with identification. The full dataset and sources for each track are provided in *SI Appendix*. Note that, in building our dataset we aimed to be inclusive and targeted a variety of tracks assigned by experts to many different dinosaur groups, but our machine learning algorithm is not informed of these group assignments. The algorithm only sees the track itself.

Each dinosaur track is represented by a two-dimensional black filled silhouette on a white background. Each image has a dimensionality of 100 × 100 pixels and silhouettes are stored in binary form, meaning each pixel has a value of 0 or 1 (see Fig. 1*A* for an exemplary image data). For training of the neural network, 96% of the data is used while the remaining 4% are reserved for validation and testing.

### Machine Learning Method.

The disentangled variational autoencoder, known as β-VAE (32), uses a neural network to encode the high-dimensional data into a low-dimensional latent space, denoted as *z*, as shown in Fig. 1. From this compressed state, the data are reconstructed by the decoder part of the network. In addition to the reconstruction loss L_rec_, the network is also evaluated based on a disentanglement criterion L_dis_, which ensures that an independent variation in the raw input data be reflected in one specific component of the compressed state, resulting in an interpretable representation of the data. This contribution to the overall loss L_all_ is balanced by a scaling factor β:

$$L_{\mathrm{all}}=L_{\mathrm{rec}}+\beta L_{\mathrm{dis}}.$$

Finding the optimum β value is challenging (32,33, 34), reflecting the question of whether the reconstruction quality and the interpretability of the created features are satisfactory. The disentanglement tries to have the features vary independently. Compared to PCA, a popular method in paleontology for distilling large datasets into more manageable components, where often components are plotted versus each other to show the distributions of different classes of the raw data, the β-VAE features should always show a 2D Gaussian distribution on these maps (*SI Appendix*, Fig. S3). The neural network is trained solely on the raw data without any a priori knowledge; that is, the features are created without any label or supervision. While the trade-off between reconstruction and disentanglement is reflected in β, an additional effect of β is the number of created features. To prioritize the reconstruction quality, in our case, a β value of 1 might be suitable. However, this creates more than 30 features which are useful for specific characterizations of the footprint—a number that we feel is too large, as many of these are subtle variations which do not appear to have anatomical or biological consequence. Here, we aim to focus on the extraction of the underlying core principle—the subset of biologically and anatomically meaningful features in which the footprints vary. Through sensitivity analyses, we find that this is best achieved by tuning β to 15, improving disentanglement and decreasing the number of key footprint features to eight, after two augmentation features are removed (Fig. 1*C*). All reconstructions shown in this study are performed on unseen test data which was not part of the training process.

### Sensitivity Analyses.

A challenge is the limited number of available samples. The task of intelligent compression and effective reconstruction necessitates complex networks with numerous free parameters, which must be learned through an optimization procedure. If such networks are trained with small datasets, they tend to memorize the data—they perform perfectly on the training data, while proving ineffective when applied to unseen validation and test data. To overcome this obstacle, we employed various data augmentation techniques. In short, the silhouette of the footprint was randomly rotated, mirrored, stretched, or compressed, a toe or a section of the track was removed, and the edge of the footprint was randomly displaced by a small number of pixels. Fig. 1*D* displays reconstructions for missing parts used as input for the neural network. It is crucial to emphasize that the presented footprints are not included in the training process. The alignment of the reconstructed parts with the original ones is convincing, in that they clearly resemble silhouettes of real footprints, demonstrating the network’s ability to generalize. This capability could be particularly valuable for incomplete fossil tracks. The architecture of the neural network is based on dividing the image into patches and processing them via multiple convolutional layers. The motivation for this approach is to have a network that is capable of identifying complicated dependencies but at the same time have the less possible number of free parameters to learn. A detailed description of the augmentation, the neural architecture, and the training process can be found in *SI Appendix*.

To analyze the performance of the network, a subsection of the data was not used in the training process and reserved for validation and testing. This is to further assess how good the network is at analyzing data it has not seen before. *SI Appendix*, Fig. S2*A* shows the overall loss during the training process for the training and test data. The generalization of the network can be observed since both curves are on top of each other. As the main contribution of this study is the unsupervised identification of features of maximum variation (=creation of footprint features), the average values of features for the classes “Quadrupedal,” “Ornithopod,” “Brothers’ Point,” “Theropod,” “Bird-like,” “Bird,” and “Extant” were calculated and their resulting reconstructions are shown in *SI Appendix*, Fig. S2*B* in comparison with the average image. Assuming the features represent independently varying attributes of the tracks, one should be able to create random feature combinations and use the decoder part of the network to create resulting tracks. This generative aspect of the network is shown in *SI Appendix*, Fig. S2*C* where some examples of these “fake” tracks are shown—these clearly are realistic silhouettes of what could be real dinosaur tracks, which lends credence to our approach.

### Trackmaker Identification Hypotheses.

Only after the training process (when the final network exists and is no longer modified) did we compare the created features to the human labels for each track. To do so, we classified tracks into seven groups based on expert identification in the literature: ornithopods, nonavian theropods, fossil birds, extant birds, quadrupedal dinosaurs, plus two groups of tracks whose affinities are debated: 13 tracks from the Middle Jurassic of Scotland that might belong to nonavian theropods or ornithopods, and eight tracks that might belong to fossil birds or bird-like nonavian theropods.

To summarize the total variation among trackmakers in the eight key footprint features, and to visually depict separations and overlaps among the taxonomic groups, we created a β-VAE morphospace. In data science, t-SNE is a powerful technique for visualizing high-dimensional data in a two-dimensional space, analogous to morphospace plots based on PCA commonly used in paleontology (*SI Appendix*, Fig. S3). In our case, the eight-dimensional feature space derived from the disentangled variational autoencoder is projected onto two dimensions using t-SNE, which considers all eight features during the dimensionality reduction process. Unlike PCA, which relies on linear transformations, t-SNE captures nonlinear relationships, making it particularly suitable for revealing clustering patterns in complex feature spaces.

To more directly inform trackmaker identification for the two sets of problematic tracks, we created an eight-dimensional morphospace defined by the key features identified by the neural network. We used distance measurements to make an informed hypothesis of trackmaker identity for the equivocal tracks, by comparing them to the labeled tracks to quantitatively enumerate which individual labeled tracks are most similar to the equivocal tracks. We employed two distance metrics: 1) a nearest-neighbor calculation, and 2) an average trait-based distance measurement. These metrics were calculated separately for two subsets of our entire footprint dataset, relevant to the debate at hand: the first included all nonavian theropods, ornithopods, and the problematic tracks that could belong to either group (Fig. 4 *A*–*C*); the second included all nonavian theropods, fossil, and modern birds, and the problematic tracks that could belong to either group (Fig. 4 *D*–*F*).

For both subsets, in order to provide context both distance metrics were calculated for each track in the dataset, not only the problematic tracks. For the nearest-neighbor metric, we identified the closest seven tracks to each given track, and calculated the percentage belonging to ornithopods and nonavian theropods. For the average trait-based metric, each given track was compared to the average of all nonavian theropod tracks and the average of all ornithopod tracks in trait space. For each metric, we calculated the distance of each footprint to each group. Then, we gathered these results in a histogram. If the track belongs to group A (in this case, ornithopods or birds, respectively) then the difference is negative; if it belongs to group B (in both cases, nonavian theropods), then the difference is positive. The absolute value of the difference depends on the strength of the difference. Results are shown in Fig. 4, with the problematic tracks placed onto the histograms. As a consistency check, the two distance metrics were plotted against each other (Fig. 4 *C* and *F*), showing that they correspond and thus approximating the same signal.

As not all eight features might have the same importance for the distance metrics, we utilized an autoscaling method giving greater weight to those of the eight features more important to the distance measurements, which returns similar results (*SI Appendix*, Figs. S4 and S5).

## Supplementary Material

Appendix 01 (PDF)

Dataset S01 (XLSX)

## Data Availability

Data have been deposited in GitHub (https://github.com/gregh83/DinoTracker) (26). All other data are included in the manuscript and/or supporting information. Previously published data were used for this work. [The raw data (silhouettes of footprints) were collected from various sources that have been published. A detailed list can be found in Dataset S1).]
