# Supplementary material for: Identifying variation in dinosaur footprints and classifying problematic specimens via unbiased unsupervised machine learning
Source: Proc Natl Acad Sci U S A. 2026 Jan 26;123(5):e2527222122. doi: 10.1073/pnas.2527222122 (PMC12867633; doi:10.1073/pnas.2527222122)
Supplement: Supplementary file 1 — Appendix 01 (PDF) [file pnas.2527222122.sapp.pdf]

Supplementary information (SI)

List of supplementary files

- Specimen data – 'tracks.xlsx'

Footprint silhouette sources

In total, we analyzed 1,974 track silhouettes, comprising of:

- **961 theropods** [Ada10, Alc14b, Ava12, Bar05, Bel10, Bla25, Cal91, Cas16, Cen22, Cob14, Con13, Dal00, Ell72, Ell74, Fal18, Fan13a, Fan13b, Far12, Far18, Fos00, Fos06, Fos15, Fue98, Fuj07, Gan05, Gas03, Gat99, Get15, Gie91, Gie94, Gie96, Gie01, Gie04, Gie09a, Gie09b, Had21, Her22, Hun98, Ish89, Ish10, Kel12, Kim17, Kle20, Kle22, Kub89, Lal15, Lal16, Lal22a, Lal22b, Lal22c, Lal22d, Leo80, Leo04, Leo21a, Li11, Li15, Li21, Lin03, Lin94, Loc96, Loc98a, Loc98b, Loc98c, Loc98d, Loc98e, Loc00b, Loc01, Loc02, Loc06b, Loc06c, Loc06d, Loc06e, Loc07, Loc08a, Loc08b, Loc11, Loc13, Loc14a, Loc14b, Loc14c, Loc14d, Loc14h, Loc15, Loc18a, Loc18b, Loc18c, Loc18d, Loc18e, Loc18f, Loc18g, Lü06, Luc06, Mar17, Mat03, Mat97, Mat05, Mat06, McC14a, McC14b, McC14c, Mey21, Mil15, Mil06a, Mil06b, Mor94, Mor21, Nie04, Nie16, Ols80, Per15, Pit89, Raa72, Rai96, Rau18, Raz17, Rey89, Rom20, Sal16, Sho89, Wag12, Wag16, Wag22, Wan16, Wee06, Wri98, Xin11, Xin13a, Xin13b, Xin13c, Xin13d, Xin14a, Xin14b, Xin14c, Xin14d, Xin14e, Xin14f, Xin14g, Xin14h, Xin14j, Xin14k, Xin15a, Xin15d, Xin15e, Xin16b, Xin16g, Xin17b, Xin19a, Xin21c, Xin21d, Xin21e, Zhe86],
- **17** additional theropod images [Bla25]

- **616 ornithopods** [Alc14a, Cal91, Cas13a, Cas13b, Cas20, Cro21, Cur89, Cur91, Dal13, Ell74, Far18, Fig21, Fio22, Gie91, Gie04, Gie08a, Gie08b, Gie09a, Gie09b, Had21, Hen17, Her16, Huh03, Hun98, Kim09, Kim16, Kle22, Kub89, Lal16, Lal22a, Leo21a, Leo21b, Li12, Li15, Lim12, Loc87, Loc95, Loc98a, Loc00a, Loc00b, Loc01, Loc02, Loc03, Loc04, Loc06d, Loc06e, Loc09, Loc14b, Loc14d, Loc14e, Loc14f, Loc14g, Loc14i, Loc18a, Loc18d, Loc18e, Loc18g, Loc21a, Lul53, Mat03, Mat05, Mey03, Mil06a, Mor92, Mor94, Nie16, Ols80, Ols03, Pas09, Per97, Raz16, Rod12, Sal16, San13, Thu94, Tsu18, Wil09, Woo89, Xin09, Xin13c, Xin14b, Xin14i, Xin15b, Xin15c, Xin15d, Xin15e, Xin16a, Xin16b, Xin16c, Xin16d, Xin16e, Xin16f, Xin16h, Xin17a, Xin21f, Xin21g, Yoo21],
- **46** additional ornithopod images [Cas22, Día16, Día24, Die04, Enr22, Gar23, Hor16, Lee18, Li23, Loc12, Mat08, Niu23, Pan24, Pon14, Sar74, Sar98, Shi19, Vil23, Xin21b, Xin25, Xin25b]

- **117 extant birds** (some collected by us) [Ara15, Elb01, Kan21]
- **98 extinct birds** from Cretaceous and Cenozoic [Abb23, Ara15, Ast16, Azu02, Car23, Doy00, Fio11, Fle23, He13, Ima18, Kan21, Kim06, Kim12, Kim 13, Li05, Lin23, Loc06, Loc07, Loc15, Loc21, Mar23, Mus12, Pat04, Rey01, Ser22, Suá16, Xin11, Xin13, Xin14, Xin15, Xin16, Xin18].

- **94 quadrupedal dinosaurs** consisting of **sauropods** [Bla25, Cas12, Dia18, Far12, Hal16, Loc94, Kim12b, Mar10, Mor19, Rig15, Sal16, San09, Xin15b], **stegosaurs** [Bel10, dePo20, Gal17, Gie08, Mat11, Mat10, Mil09, Ouc22, Pas12, Why94, Xin13b, Xin15c, Xin19] and **ankylosaurs** [Ant23, Fra18, Hor14, Loc06b, Loc14, Loc14b, Loc18, Mcc14, Pet20, Pon14, Ros18]

- **7 bird-like tracks** [Abr17, Abr23, Wee18]
- **4 unlabelled** (originally labelled 'bird-like') [Gie17]
- **14 tridactyl theropod-or-ornithopod** silhouettes representing 13 tracks found at Brother's Point on the Isle of Skye [dePo20, And84]

Each track silhouette is listed in a spreadsheet – 'tracks.xlsx' – in which we state: 'ID' – track silhouette file name; 'Group' – respective trackmaker; 'Reference' – silhouette source (which can be used to cross-reference with the 'Track references' reference list file); 'Ichnotaxon or species' – if stated; 'Period' – age of track given in epochs; and 'Fig. No.' – figure (or page number) representing the track from their respective publications (if stated) optionally with description for

track if multiple are present. For ease of navigation, we accompany this spreadsheet with a 'track reference list' (see below) with DOIs (when available). This combines our silhouette sources with those originally used by Lallensack *et al*. (2022). The track silhouettes are provided in the 'track silhouettes.zip' folder as .png files, which contains subfolders with respective tracks made by: **theropods** (untrained data within separate subfolder), **ornithopods** (untrained data within separate subfolder), **quadrupedal dinosaurs** (including sauropods, stegosaurs, and ankylosaurs), **extinct birds**, **extant birds**, **bird-like**, **unlabelled,** and **BP** (tridactyl tracks found at Brother's Point on the Isle of Skye).

Augmentation and training

- **Random silhouette edge displacement**: The edge of the silhouette, drawn by a domain expert, is randomly displaced by 1-5 pixels in a random direction. This accounts for variance in how different experts may draw the outline.
- **Horizontal flipping**: The track is mirrored horizontally representing creation by either the left or right foot.
- **Vertical and horizontal stretching and compression**: The track is stretched or compressed by 0-3 pixels, simulating potential alterations caused by taphonomy and rock deformation occurring over millions of years.
- **Toe removal**: With a 20% chance, a randomly chosen toe is removed in the input of the network while the reconstruction target still includes all toes.
- **Circle removal**: Similar to toe removal, and also occurring at a frequency of 20%, a randomly placed circle is removed from the footprint. This reflects the concept that parts of tracks may be missing and do not necessarily have to constitute an entire toe.
- **Rotation:** The image is rotated by a random angle from -30 to 30 degrees.

These augmentations are applied to the raw data to significantly increase the quantity of training data. The test data, which the network doesn't utilize during the training process and is reserved for validating the network's performance, is not subjected to augmentation. Fig. S1 illustrates some of the augmentations applied to the same raw data image. The network learns the augmentation of mirroring and rotating in multiple features (Fig. 1, S1-2).

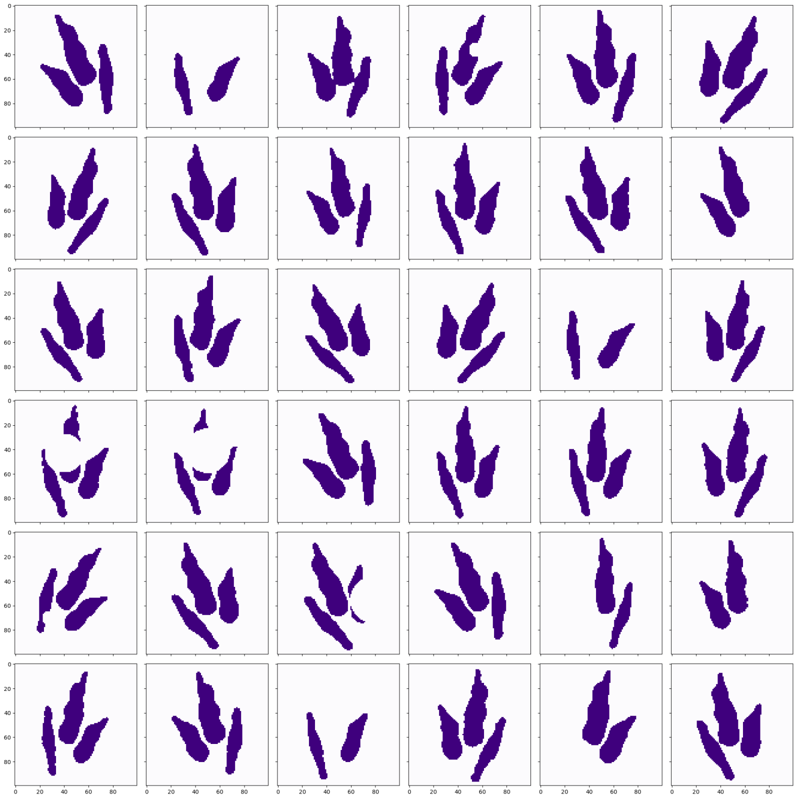

Figure S1. From one original sample multiple augmented samples are created. The toe and circle removal augmentations are indicated while the rotation, edge displacement, stretching, compression and flipping are also applied randomly to the augmented samples.

Testing process

Initially, we split our data is split into training data (96% of dataset) and validation and test data (4%). The network is then trained only with training data. Trained means that good neuron configurations are determined for the given loss function (in our case a mixture of reconstruction error and disentanglement error). In the worst-case scenario, the network entirely memorizes the training data achieving perfect reconstruction and disentanglement on the data-train while having gigantic loss values for unseen data. For that reason, we need validation data (used during training) and test data (outside the training process) which we use for the networks error on unseen data. The best-case scenario is generalization. This means that the network learns only general aspects from training data. We can check if either of the cases appear by looking at the loss value over epoch (epoch is one optimization step of the network's neurons). In Fig 2a one can see that we are in the best-case scenario because the loss of the training and test data is almost identical (and not training loss decreasing while test loss increasing).

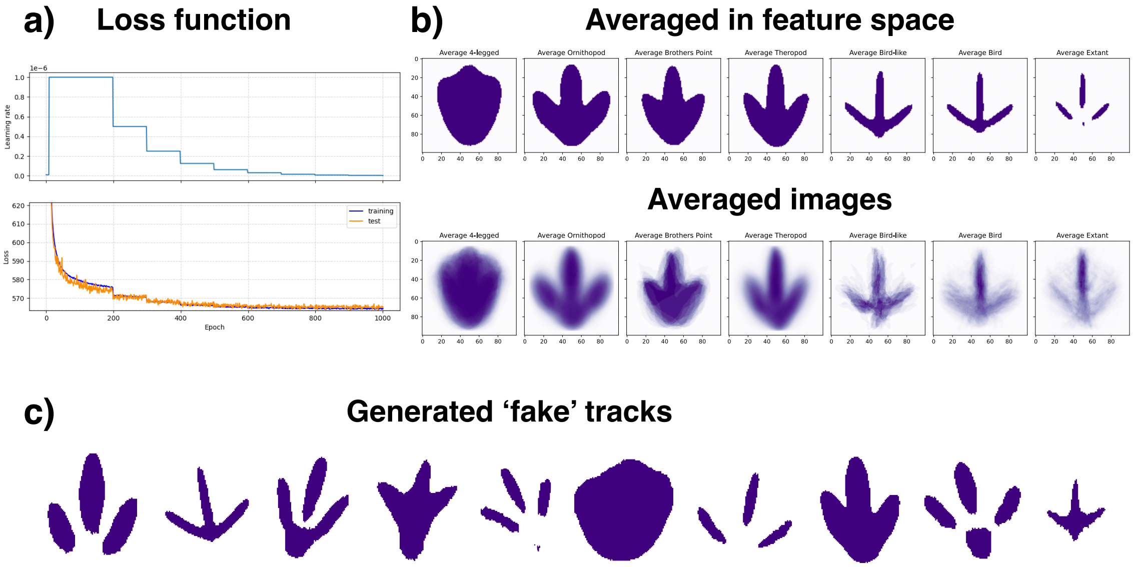

Figure S2. Analysis of the network’s performance: Over the course of training, the network achieves similar values of the overall loss, consisting of the sum of the disentanglement and reconstruction error, for the training and the unseen test data (a). For the different classes, the average image as well as the average feature values with their corresponding reconstruction is shown (b). Showcasing the successful disentanglement, one can create random feature values and use the decoder to create realistic fake tracks (c).

Neural architecture

The input data is divided into 10*10 patches [CNN22]. The encoder part of the network consists of multiple convolutional layers, with average pooling performed after mish-activation [MIS19]. The kernel sizes are 3x3 and one-dimensional padding is applied. Following this, a multi-layer perceptron (MLP) maps the resulting reduced images to a 50-dimensional σ- and μ-vector, which performs the sampling operation to a 50-dimensional bottleneck z. The result of which is a maximum number of created features of 50. The decoder involves an MLP projecting z to multiple 5x5 images, which are then demixed [MLP21, CNN22] by multiple convolutional layers. The patches are resized to a single image with sigmoid activation, automatically giving a value range of [0,1]. In order to keep the silhouette nature of the data, a threshold is finally set while values which are below 0.5 are set to 0 and all others to 1. The reconstruction loss is calculated by measuring the squared error of the input, with the removal augmentations disabled during the network's reconstruction. The disentanglement loss is given by the Kullback-Leibler-divergence of z compared to normal distributions located at zero with a width of 1. The stochastic gradient descend (SGD) optimizer with a momentum rate of 0.8 is employed with a scheduled learning rate ranging from 1e-6 to 1e-8 and a batch size of 256. It performs slightly better than other popular optimizers for given data set and network architecture. The training lasts for 1000 epochs. The standard variation of μ is used to analyze component usage. During training, the network processes the same amount of data for all three classes “theropod”, “ornithopod” and “bird”. This is achieved by creating three data pools, each used with the same probability, from which samples are randomly chosen. The current sample is then augmented. Various different architectures and hyper parameters of the network as well as the augmentation were tested. We chose the method with the overall best performance while still having similar loss values for training and unseen test data.

PCA vs. VAE

As principal component analysis (PCA) is a common technique in paleontology, Fig. S3 shows the core differences between it and our β-VAE approach. Both methods are applied to the same data set and both use 10 components. In PCA, plotting the coordinates in the principal component space often leads to correlations which are then interpreted to see some clustering, for instance. These correlations are often also indications for non-linearities, which cannot be covered by linear PCA transformations. In β-VAE, the goal is to have the new feature space in a disentangled way meaning no dependency in feature vs feature maps. Additionally, in PCA the reconstruction of the high dimensional input data (in this case, the footprints) is suboptimal, which can be improved by the β-VAE method. Achieving both goals, a disentangled low space representation and a better reconstruction, is a difficult task. For that reason, neural networks are used for both the encoder and decoder part of the process.

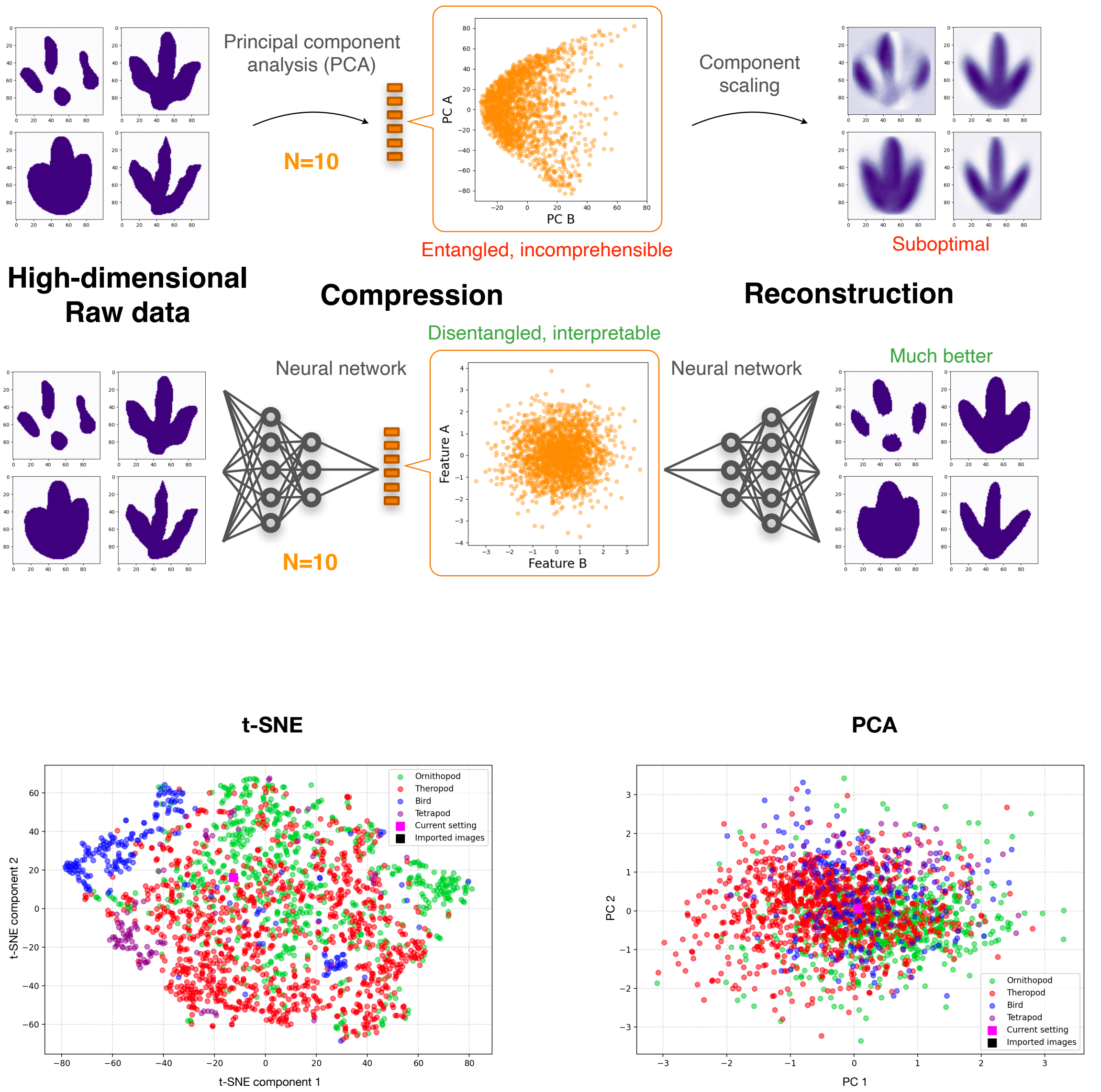

Figure S3. Comparison of how principal component analysis (a common technique in paleontology) and our neural network β-VAE approach distill multivariate data into a morphospace and a set of reconstructed tracks (above panels). As can be seen, the β-VAE approach results in a disentangled representation while also outperforming in terms of reconstruction quality. A comparison of the two-dimensional morphospaces generated by both approaches applied to our dataset (below). In both cases, the method (t-SNE or PCA) received the 8-dimensional feature space from the β -VAE as input and created a 2d morphospace from it. There is clearly more interpretable separation between footprint groups (labelled a posteriori from expert opinions) in the t-SNE approach that incorporates information from all eight axes in creating the two-dimensional morphospace as compared to the standard PCA approach that only figures the first two principal components to create a two-dimensional morphospace.

Autoscaling

In order to improve the classification performance for ornithopod vs. non-avian theropod and bird-like vs. non-avian theropod, each feature gets a scaling factor in [0,1] in the distance calculation. For both distances this is done individually. For the initial process, each factors is set to a random value and then the Basinhopping [BH12] optimizer tries to maximize the classification accuracy. To balance the classification performance the minimum of both classification accuracies is used as the optimization target. This is repeated multiple times. Results are given in Figs. S4-5.

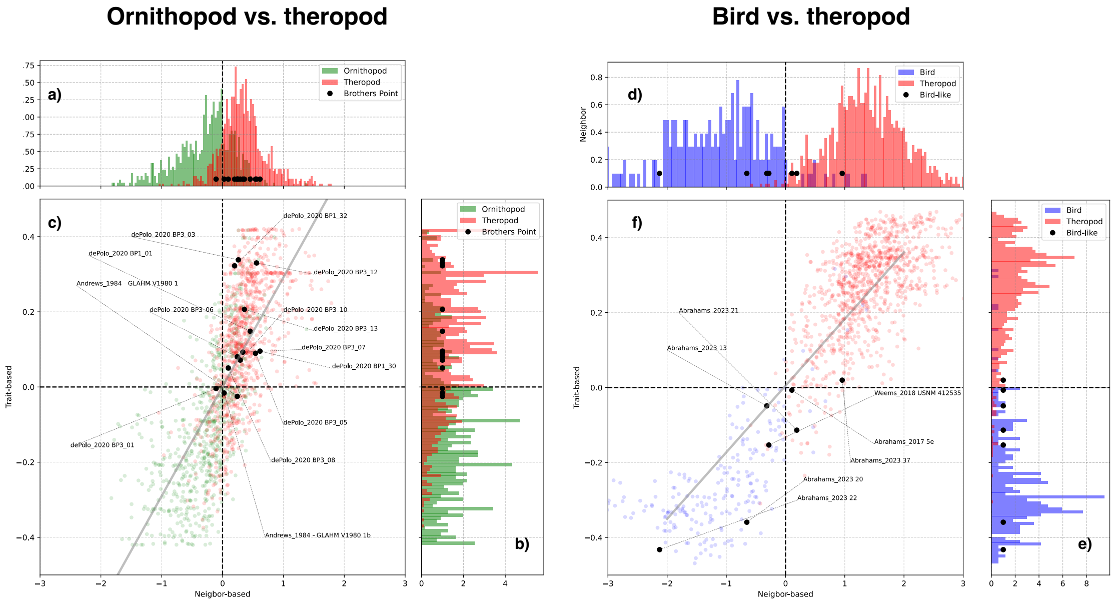

Figure S4. Distance measurements for identifying problematic tracks, as in Figure 4, but the distances here calculated with our autoscaling procedure.

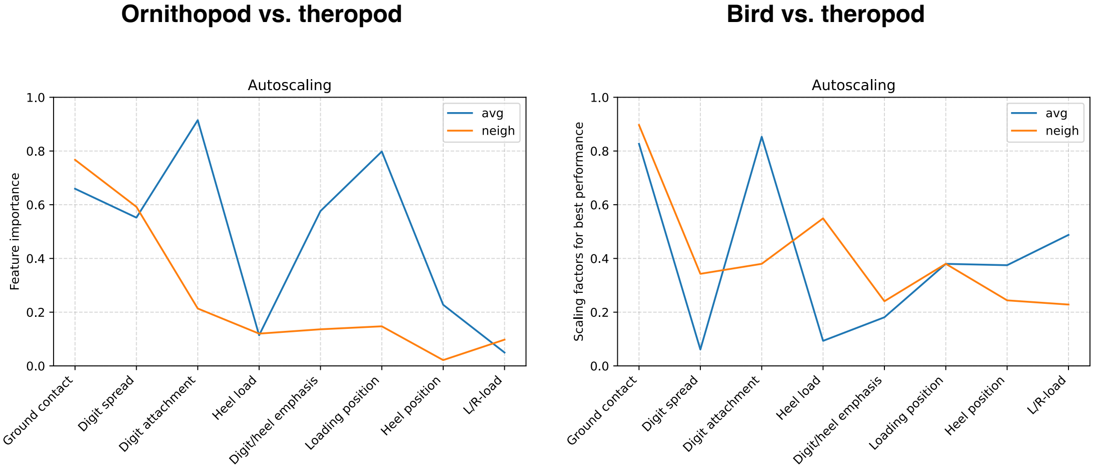

Figure S5: Determined scaling factors reflection the feature importance for both classification tasks.

The DinoTracker App (v1.0)
The model, the data pool, the source code for the app, installation instructions, the creation of training data and the training process of the network can be found at:

https://github.com/gregh83/DinoTracker
(release “v1.0”)

The app is free to download and use for all. We have designed it so that it will be especially useful to researchers who discover new dinosaur tracks, as they can simply input a silhouette of their track(s) into the app and then the app will identify the most similar tracks to the new track(s), and allow the user to understand how the eight key features of footprint variation are expressed in their new track(s). This information can be marshalled by the user to help understand how their track(s) compare to other tracks, and thus provide an informed trackmaker identification for their tracks(s).

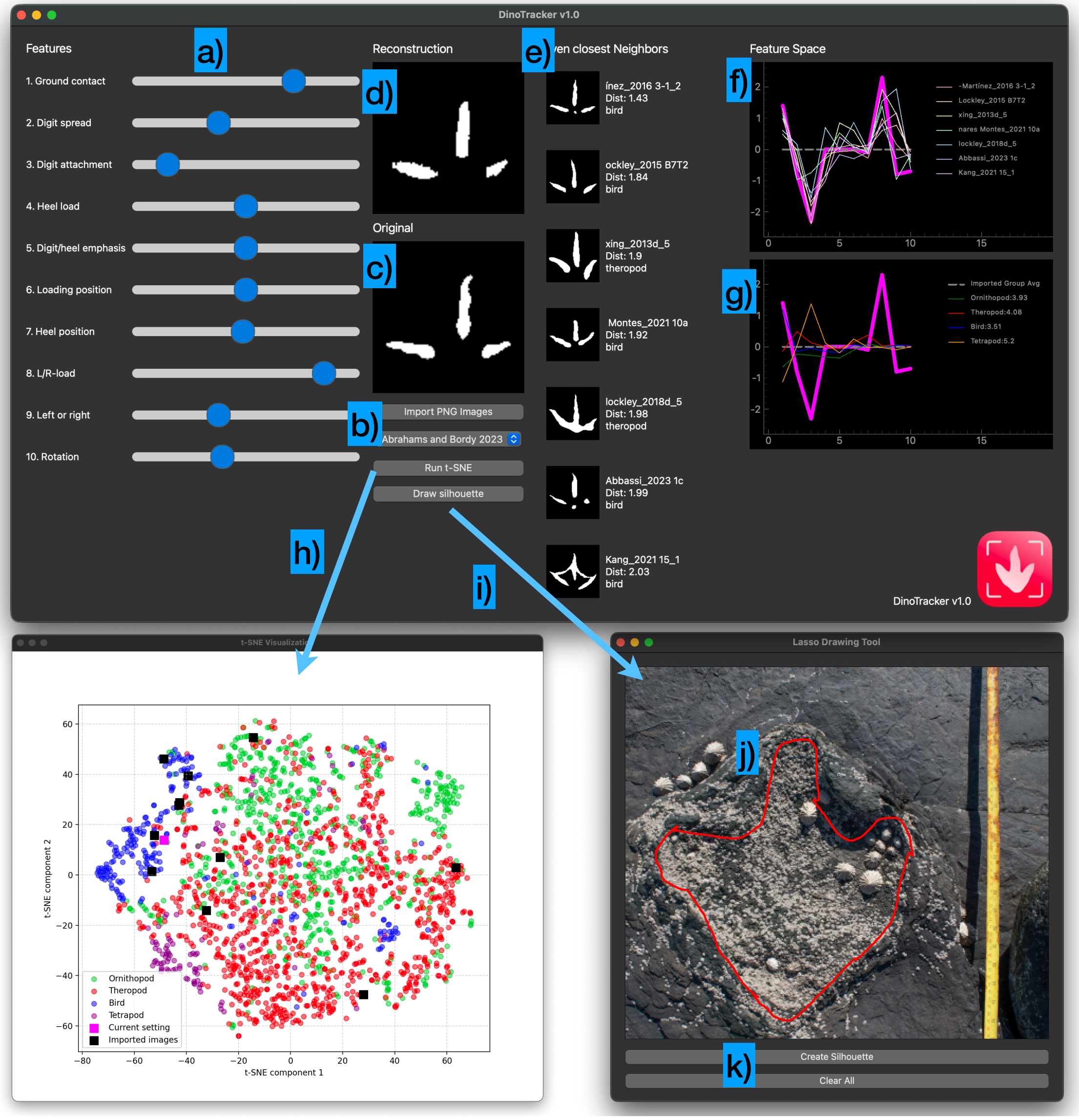

Figure S6: Screenshot of the app DinoTracker, showing its functionality when a new track silhouette is input by the user as well as the t-SNE and drawing pop-up windows.

Figure S6 shows the graphical user interface of the app alongside the two pop-up windows for t-SNE calculation and silhouette drawing, which will be explained in detail in the following.

a) The feature space is visualized with 10 sliders that can also be manipulated to explore the change in the reconstruction (d).
b) Silhouettes can be imported via file browser and, in case multiple tracks are imported, one can select one image from the group of imports.
c) The imported and currently selected original image is displayed.
d) The corresponding reconstruction is plotted.
e) For the selected silhouette, the 7 closest neighbors are displayed with their name, classification in literature and distance in feature space.
f) The feature space of the selected silhouette and its closest neighbors is shown to explain the neighbor-based distance.
g) The selected sample is compared to the average representation of each class to have a visualization of the trait-based distance. Additionally, the average representation of all imported footprints is displayed.
h) The t-SNE map is first calculated (this can take up to a minute) and then plotted in a separate window. The calculation is performed for the imported group as well as the currently selected sample.
i) In order to create a silhouette from a given photograph of a track, one can import an image and then draw a silhouette.
j) The best experience is given here when using a tablet with an electronic pencil.
k) The drawn silhouette can then be exported. In this process the silhouette is cropped to match the setting where the network was trained.

References

[BH12]: Olson, B., Hashmi, I., Molloy, K., and Shehu1, A., Basin Hopping as a General and Versatile Optimization Framework for the Characterization of Biological Macromolecules, Advances in Artificial Intelligence, Volume 2012 (2012), Article ID 674832, DOI:10.1155/2012/674832

[MLP21]:Ilya Tolstikhin and Neil Houlsby and Alexander Kolesnikov and Lucas Beyer and Xiaohua Zhai and Thomas Unterthiner and Jessica Yung and Andreas Steiner and Daniel Keysers and Jakob Uszkoreit and Mario Lucic and Alexey Dosovitskiy: MLP-Mixer: An all-MLP Architecture for Vision, year={2021},eprint={2105.01601}, archivePrefix={arXiv}.

[CNN22]: Asher Trockman and J. Zico Kolter: Patches Are All You Need?, year={2022}, https://doi.org/10.48550/arXiv.2201.09792

Track reference list

All the track silhouettes used in this study are referenced below. Each reference is accompanied by their respective abbreviation (highlighted in bold) to ease cross-referencing with trackmaker categories in the ‘footprint silhouette source’ section.

[Abb23] Abbassi, N., Gasparik, M., Kordos, L. and Esmaeili, F., 2023. Evaluation of *Iranipeda abeli* (Avian track) from the Zagros Mountains, SW Iran: re-examination of the original material and overview of primary sources. *Historical Biology*, *36*(3), pp.562-571. doi: 10.1080/08912963.2023.2173071

[Abr17] Abrahams, M., Bordy, E.M., Sciscio, L. and Knoll, F., 2017. Scampering, trotting, walking tridactyl bipedal dinosaurs in southern Africa: ichnological account of a Lower Jurassic

palaeosurface (upper Elliot Formation, Roma Valley) in Lesotho. *Historical Biology*, *29*(7), pp.958-975. doi: 10.1080/08912963.2016.1267164

[Abr23] Abrahams, M. and Bordy, E.M., 2023. The oldest fossil bird-like footprints from the upper Triassic of southern Africa. *Plos one*, *18*(11), p.e0293021. doi: 10.1371/journal.pone.0293021

[Ada10] Adams, T.L., Strganac, C., Polcyn, M.J. and Jacobs, L.L., 2010. High resolution three-dimensional laser-scanning of the type specimen of Eubrontes (?) glenrosensis Shuler, 1935, from the Comanchean (Lower Cretaceous) of Texas: implications for digital archiving and preservation. *Palaeontologia Electronica*, *13*(3), pp. 1-11.

[Alc14a] Alcalá, L., Mampel, L., Royo-Torres, R. and Cobos, A., 2014a. On small quadrupedal ornithopod tracks in Jurassic-Cretaceous transition intertidal deposits (El Castellar, Teruel, Spain). *Spanish Journal of Palaeontology*, *29*(2), pp.183-190. doi: 10.7203/sjp.29.2.17800

[Alc14b] Alcalá, L., Pérez-Lorente, F., Luque, L., Cobos, A., Royo-Torres, R. and Mampel, L., 2014b. Preservation of dinosaur footprints in shallow intertidal deposits of the Jurassic-Cretaceous transition in the Iberian Range (Teruel, Spain). *Ichnos*, *21*(1), pp.19-31. doi: 10.1080/10420940.2013.873721

[And84] Andrews, J.E. and Hudson, J.D., 1984. First Jurassic dinosaur footprint from Scotland. *Scottish Journal of Geology*, *20*(2), pp.129-134. doi: 10.1144/sjg20020129

[Ant23] Antonelli, M., Petti, F.M., Conti, J., Sacco, E., Petruzzelli, M., Spalluto, L. and Wagensommer, A., 2023. Lower Cretaceous dinosaur footprints from the Molfetta tracksite (Apulia, southern Italy). *Cretaceous Research*, *142*, p.105388. doi: 10.1016/j.cretres.2022.105388

[Ara15] Aramayo, S.A., de Bianco, T.M., Bastianelli, N.V. and Melchor, R.N., 2015. Pehuen Co: Updated taxonomic review of a late Pleistocene ichnological site in Argentina. *Palaeogeography, Palaeoclimatology, Palaeoecology*, *439*, pp.144-165. doi: 10.1016/j.palaeo.2015.07.006

[Ast17] Astibia, H., Rodríguez-Tovar, F.J., Díaz-Martínez, I., Payros, A. and Ortiz, S., 2017. Trace fossils from the Middle and Upper Eocene (Bartonian–Priabonian) molasse deposits of the Pamplona Basin (Navarre, western Pyrenees): palaeoenvironmental implications. *Geological Journal*, *52*(2), pp.327-349. doi: 10.1002/gj.2763

[Ava12] Avanzini, M., Piñuela, L. and García-Ramos, J.C., 2012. Late Jurassic footprints reveal walking kinematics of theropod dinosaurs. *Lethaia*, *45*(2), pp.238-252. doi: 10.1111/j.1502-3931.2011.00276.x

[Azu02] Azuma, Y., Arakawa, Y., Tomida, Y., and Currie, P.J. 2002. Early Cretaceous bird tracks from the Tetori Group, Fukui Prefecture, Japan. *Memoir of the Fukui Prefectural Dinosaur Museum*, *1*, pp.1-6.

[Bar05] Barco, J.L., 2005. Evidencia icnológica de un dinosaurio terópodo gigante en el Berriasiense (Cretácico Inferior) de Laurasia (Las Villasecas, Soria, España). *Revista española de paleontología*, *10*, pp.59-71.

[Bel10a] Belvedere, M., Mietto, P. and Ishigaki, S., 2010. A Late Jurassic diverse ichnocoenosis from the siliciclastic Iouaridene formation (Central High Atlas, Morocco). *Geological Quarterly*, *54*(3), pp.367-380.

[Bel10b] Belvedere, M. and Mietto, P., 2010. First evidence of stegosaurian Deltapodus footprints in North Africa (Iouaridène Formation, Upper Jurassic, Morocco). *Palaeontology*, *53*(1), pp.233-240. doi: 10.1111/j.1475-4983.2009.00928.x

[Bla25] Blakesley, T., dePolo, P.E., Wade, T.J., Ross, D.A. and Brusatte, S.L., 2025. A new Middle Jurassic lagoon margin assemblage of theropod and sauropod dinosaur trackways from the Isle of Skye, Scotland. *PLoS One*, *20*(4), p.e0319862. doi: 10.1371/journal.pone.0319862

[Cal91] Calvo, J.O. 1991. Huellas de dinosaurios en la Formación Río Limay (AlbianoCenomaniano?), Picún Leufú, provincia de Neuquén, República Argentina. (OrnithischiaSaurischia: Sauropoda-Theropoda). Ameghiniana, 28, pp. 241–258.

[Cas12] Castanera, D., Pascual, C., Canudo, J.I., Hernandez, N. and Barco, J.L., 2012. Ethological variations in gauge in sauropod trackways from the Berriasian of Spain. *Lethaia*, *45*(4), pp.476-489. doi: 10.1111/j.1502-3931.2012.00304.x

[Cas13a] Castanera, D., Pascual, C., Razzolini, N.L., Vila, B., Barco, J.L. and Canudo, J.I., 2013a. Discriminating between medium-sized tridactyl trackmakers: tracking ornithopod tracks in the base of the Cretaceous (Berriasian, Spain). PloS one, 8(11), p.e81830. doi: 10.1371/journal.pone.0081830

[Cas13b] Castanera, D., Vila, B., Razzolini, N.L., Falkingham, P.L., Canudo, J.I., Manning, P.L. and Galobart, A., 2013b. Manus track preservation bias as a key factor for assessing trackmaker identity and quadrupedalism in basal ornithopods. PloS one, 8(1), p.e54177. doi: 10.1371/journal.pone.0054177

[Cas16] Castanera, D., Santos, V.F., Piñuela, L., Pascual, C., Vila, B., Canudo, J.I. and Moratalla, J.J. 2016. Iberian sauropod tracks through time: variations in sauropod manus and pes track morphologies. In Falkingham, P.L., Marty, D. and Richter, A. (eds.) Dinosaur Tracks: The Next Steps. Bloomington: Indiana University Press. pp. 120–137.

[Cas20] Castanera, D., Silva, B.C., Santos, V.F., Malafaia, E., and Belvedere, M. 2020. Tracking Late Jurassic ornithopods in the Lusitanian Basin of Portugal: Ichnotaxonomic implications. Acta Palaeontologica Polonica 65 (2): pp. 399-412. doi: 10.4202/app.00707.2019

[Cas22] Castanera, D., Bádenas, B., Aurell, M., Canudo, J.I. and Gasca, J.M., 2022. New ornithopod tracks from the Lower Cretaceous El Castellar Formation (Spain): Implications for track preservation and evolution of ornithopod footprints. *Palaeogeography, Palaeoclimatology, Palaeoecology*, *591*, p.110866. doi: 10.1016/j.palaeo.2022.110866

[Cen22] Ceniceros, J.M., Farlow, J.O., Masrour, M., Extremiana, J.I., Boutakiout, M. and Perez-Lorente, F., 2022. Demographic interpretation of colossal theropod footprints discoveries from Imilchil (Mid-Jurassic, Central High Atlas, Morocco). *Journal of African Earth Sciences*, *193*, p.104595. doi: 10.1016/j.jafrearsci.2022.104595

[Cob14] Cobos, A., Lockley, M.G., Gasco, F., Royo–Torres, R. and Alcala, L., 2014. Megatheropods as apex predators in the typically Jurassic ecosystems of the Villar del Arzobispo Formation (Iberian Range, Spain). *Palaeogeography, Palaeoclimatology, Palaeoecology*, *399*, pp.31-41. doi: 10.1016/j.palaeo.2014.02.008

[Con13] Contessi, M. 2013. A new dinosaur ichnofauna from Tunisia: Implications for the palaeobiogeography of peri-Adriatic carbonate platforms in the mid-Cretaceous. *Palaeogeography, Palaeoclimatology, Palaeoecology*, 392, pp.302–311. doi: 10.1016/j.palaeo.2013.09.018

[Cro21] Crowell, J. K. and Shimer, G. T. 2021. Late Cretaceous Dinosaur Tracks from the Iron Springs Formation, Iron County, Utah. *The Compass: Earth Science Journal of Sigma Gamma Epsilon*, 91, 1. pp.1-38. doi: 10.62879/c59475171

[Cur89] Currie, P. J. 1989. Dinosaur footprints of western Canada. *International symposium on dinosaur tracks and traces*. 1, pp.293–300.

[Cur91] Currie, P.J. Nadon, G.C. and Lockley, M.G. 1991. Dinosaur footprints with skin impressions from the Cretaceous of Alberta and Colorado. *Canadian Journal of Earth Sciences*, 28, pp.102–115. doi: doi.org/10.1139/e91-009

[Dal00] Dalla Vecchia, F.M., Tarlao, A., Tunis, G. and Venturini, S. 2000. New dinosaur tracksites in the Albian (Early Cretaceous) of the Istrian Peninsula (Croatia)— Part II—Paleontology. *Memorie di Scienze Geologiche*, 52, pp.227–292.

[Dal13] Dalman, S.G. and Weems, R.E. 2013. A new look at morphological variation in the ichnogenus Anomoepus, with special reference to material from the Lower Jurassic Newark Supergroup: implications for ichnotaxonomy and ichnodiversity. *Bulletin of the Peabody Museum of Natural History*, 54, pp.67–124. doi: 10.3374/014.054.0104

[deP20] dePolo, P.E., Brusatte, S.L., Challands, T.J., Foffa, D., Wilkinson, M., Clark, N.D., Hoad, J., Pereira, P.V.L.G.D.C., Ross, D.A. and Wade, T.J., 2020. Novel track morphotypes from new tracksites indicate increased Middle Jurassic dinosaur diversity on the Isle of Skye, Scotland. *PLoS One*, *15*(3), p.e0229640. doi: 10.1371/journal.pone.0229640

[Día16a] Díaz-Martínez, I., de Valais, S. and Cónsole-Gonella, C., 2016a. First evidence of Hadrosauropodus in Gondwana (Yacoraite Formation, Maastrichtian-Danian), northwestern Argentina. *Journal of African Earth Sciences*, *122*, pp.79-87. doi: 10.1016/j.jafrearsci.2016.02.012

[Día16b] Díaz Martínez, I., Suarez Hernando, O., Martínez García, B.M., Larrasoaña, J.C. and Murelaga, X., 2016b. First bird footprints from the lower Miocene Lerín Formation, Ebro Basin, Spain. *Palaeontologia Electronica*, 19.1.7A, pp.1-15. doi: 10.26879/604

[Día18] Díaz-Martínez, I., de Valais, S. and Console-Gonella, C., 2018. New sauropod tracks from the Yacoraite Formation (Maastrichtian–Danian), Valle del Tonco tracksite, Salta, northwestern Argentina. *Journal of Iberian Geology*, *44*(1), pp.113-127. doi: 10.1007/s41513-017-0035-1

[Día24] Díaz-Martínez, I., Citton, P. and Castanera, D., 2024. What do their footprints tell us? Many questions and some answers about the life of non-avian dinosaurs. *Journal of Iberian Geology*, *50*(1), pp.5-26. doi: 10.1007/s41513-023-00226-6

[Die04] Diedrich, C., 2004. New important iguanodontid and theropod trackways of the tracksite Obernkirchen in the Berriasian of NW Germany and megatracksite concept of Central Europe. *Ichnos*, *11*(3-4), pp.215-228. doi: 10.1080/10420940490444924

[Doy00] Doyle, P., Wood, J.L. and George, G.T., 2000. The shorebird ichnofacies: an example from the Miocene of southern Spain. *Geological Magazine*, *137*(5), pp.517-536. doi: 10.1017/S0016756800004490

[Elb01] Elbroch, M. and Marks, E., 2001. *Bird tracks & sign: a guide to North American species.* Mechanicsburg: Stackpole Books.

[Ell72] Ellenberger, P. 1972. Contribution à la classification des pistes de vertébrés du Trias: Les types du Stormberg d´Afrique du Sud (I). *Palaeovertebrata, Memoire Extraordinaire*, pp.1–152.

[Ell74] Ellenberger, P. 1974. Contribution à la classification des pistes de vertébrés du Trias: Les types du Stormberg d´Afrique du Sud (II éme partie: Le Stormberg Superieur - I. Le biome de la zona B/1 ou niveau de Moyeni: ses biocénoses). *Palaeovertebrata, Memoire Extraordinaire*, pp.1–143.

[Enr22] Enriquez, N.J., Campione, N.E., White, M.A., Fanti, F., Sissons, R.L., Sullivan, C., Vavrek, M.J. and Bell, P.R., 2022. The dinosaur tracks of tyrants aisle: An Upper Cretaceous ichnofauna from unit 4 of the Wapiti Formation (upper Campanian), Alberta, Canada. *Plos one*, *17*(2), p.e0262824. doi: 10.1371/journal.pone.0262824

[Eva16] Evans, J. 2016 *Bird Tracks and Sign*. Available at: https://naturetracking.com/bird-tracks/ (Accessed: 12th August 2025).

[Fal18] Falkingham, P.L., Bates, K.T., Avanzini, M., Bennett, M., Bordy, E.M., Breithaupt, B.H., Castanera, D., Citton, P., Díaz-Martínez, I., Farlow, J.O., Fiorillo, A.R., Gatesy, S.M., Getty, P., Hatala, K.G., Hornung, J.J., Hyatt, J.A., Klein, H., Lallensack, J.N., Martin, A.J., Marty, D., Matthews, N.A., Meyer, C.A., Milàn, J., Minter, N.J., Razzolini, N.L., Romilio, A., Salisbury, S.W., Sciscio, L., Tanaka, I., Wiseman, A.L.A., Xing, L.D. and Belvedere, M. 2018. A standard protocol for documenting modern and fossil ichnological data. *Palaeontology*, 61, pp.469–480. doi: 10.1111/pala.12373

[Fan13a] Fanti, F., Bell, P.R. and Sissons, R.L. 2013a. A diverse, high-latitude ichnofauna from the Late Cretaceous Wapiti Formation, Alberta, Canada. *Cretaceous Research*, 41, pp.256–269. doi: 10.1016/j.cretres.2012.12.010

[Fan13b] Fanti, F., Contessi, M., Nigarov, A. and Esenov, P. 2013b. New Data on Two Large Dinosaur Tracksites from the Upper Jurassic of Eastern Turkmenistan (Central Asia). *Ichnos*, 20, pp.54–71. doi: 10.1080/10420940.2013.778845

[Far12] Farlow, J.O., O'Brien, M., Kuban, G.J., Dattilo, B.F., Bates, K.T., Falkingham, P.L., Piñuela, L., Rose, A., Freels, A., Kumagai, C., Libben, C., Smith, J. and Whitcraft, J. 2012. Dinosaur Tracksites of the Paluxy River Valley (Glen Rose Formation, Lower Cretaceous), Dinosaur Valley State Park, Somervell County, Texas. In *Actas de V Jrnadas Internacionales sobre Paleontologia de Dinosaurios y su Entorno, Salas de los Infantes, Burgos.* pp.41-69.

[Far18] Farlow, J.O., Coroian, D. and Currie, P.J. 2018. Noah's Ravens: Interpreting the Makers of Tridactyl Dinosaur Footprints. Life of the Past. Bloomington: Indiana University Press.

[Fig21] Figueiredo, S.D., de Carvalho, C.N., Cunha, P.P. and de Sousa Carvalho, I., 2021. New Dinosaur Tracks from the Lower Barremian of Portugal (Areia do Mastro Formation, Cape Espichel). *Journal of Geoscience and Environment Protection*, 9 (1), pp. 84-96. doi: 10.4236/gep.2021.91007

[Fig22] Figueiredo, S.D., Cunha, P.P., Suberbiola, X.P., de Carvalho, C.N., de Souza Carvalho, I., Buffetaut, E., Tong, H., Sousa, M.F., Antunes, V. and Anastácio, R., 2022. The dinosaur tracksite from the lower Barremian of Areia do Mastro Formation (Cabo Espichel, Portugal): implications for dinosaur behavior. *Cretaceous Research*, *137*, p.105219. doi: 10.1016/j.cretres.2022.105219

[Fio11] Fiorillo, A.R., Hasiotis, S.T., Kobayashi, Y., Breithaupt, B.H. and McCarthy, P.J., 2011. Bird tracks from the Upper Cretaceous Cantwell Formation of Denali National Park, Alaska, USA: a new perspective on ancient northern polar vertebrate biodiversity. *Journal of Systematic Palaeontology*, *9*(1), pp.33-49. doi: 10.1080/14772019.2010.509356

[Fle23] Fleury, K., Burns, E., Richards, M.D., Norton, K., Read, S., Wesley, R., Ewan Fordyce, R. and Wilcken, K., 2023. The moa footprints from the Pliocene–early Pleistocene of Kyeburn, Otago, New Zealand. *Journal of the Royal Society of New Zealand*, *54*(5), pp.620-642. doi: 10.1080/03036758.2023.2264789

[Fos00] Foster, J.R., Hamblin, A.H. and Lockley, M.G., 2000. The oldest evidence of a sauropod dinosaur in the western united states and other important vertebrate trackways from grand

staircase-escalante national monument, Utah. Ichnos: An International Journal of Plant & Animal, 7(3), pp.169-181. doi: 10.1080/10420940009380158

[Fos06] Foster, J.R. and Lockley, M.G. 2006. The vertebrate ichnological record of the Morrison Formation (Upper Jurassic, North America). In Foster, J.R. and Lucas, S.G. (eds.) Paleontology and Geology of the Upper Jurassic Morrison Formation, Vol. 36. 203–216 pp.

[Fos15] Foster, J.R., 2015. Theropod dinosaur ichnogenus *Hispanosauropus* identified from the Morrison Formation (Upper Jurassic), western North America. *Ichnos*, 22 (3-4), pp.183-191. doi: 10.1080/10420940.2015.1059335

[Fra18] Francischini, H., Sales, M.A., Dentzien–Dias, P. and Schultz, C.L., 2018. The presence of Ankylosaur tracks in the Guará Formation (Brazil) and remarks on the spatial and temporal distribution of Late Jurassic Dinosaurs. *Ichnos*, 25 (2-3), pp.177-191. doi: 10.1080/10420940.2017.1337573

[Fue98] Fuentes Vidarte, C. and Meijide Calvo, M., 1998. Icnitas de dinosaurios terópodos en el Weald de Soria (España). Nuevo icnogénero Kalohipus. *Estudios geológicos*, 54 (3-4), pp.147-152. doi: 10.3989/egeol.98543-4213

[Fuj07] Fujita, M., Azuma, Y., Lee, Y.N., Lü, J., Dong, Z., Noda, Y. and Urano, K., 2007. New theropod track site from the Upper Jurassic Tuchengzi Formation of Liaoning Province, northeastern China. *Memoir of the Fukui Prefectural Dinosaur Museum*, 6, pp.17-25.

[Gal17] Galton, P.M. and Ayyasami, K., 2017. Purported latest bone of a plated dinosaur (Ornithischia: Stegosauria), a “dermal plate” from the Maastrichtian (Upper Cretaceous) of southern India. *Neues Jahrbuch für Geologie und Paläontologie-Abhandlungen*, 285 (1), pp.91-96. doi: 10.1127/njgpa/2017/0671

[Gan05] Gand, G., Demathieu, G., Grancier, M. and Sciau, J., 2005. Les traces dinosauroïdes du Trias supérieur français: discrimination, interprétation et comparaison. *Bulletin de la Société géologique de France*, *176*(1), pp.69-79.

[Gar23] García-Cobeña, J., Cobos, A. and Verdú, F.J., 2023. Ornithopod tracks and bones: Paleoecology and an unusual evidence of quadrupedal locomotion in the Lower Cretaceous of eastern Iberia (Teruel, Spain). *Cretaceous Research*, *144*, p.105473. doi: 10.1016/j.cretres.2023.105473

[Gas03] Gaston, R., Lockley, M.G., Lucas, S. and Hunt, A., 2003. *Grallator*-dominated fossil footprint assemblages and associated enigmatic footprints from the Chinle Group (Upper Triassic), Gateway area, Colorado. *Ichnos*, 10 (2-4), pp.153-163. doi: 10.1080/10420940390256258

[Gat99] Gatesy, S.M., Middleton, K.M., Jr, F.A.J. and Shubin, N.H. 1999. Three-dimensional preservation of foot movements in Triassic theropod dinosaurs. *Nature*, 399, pp.141-144. doi: 10.1038/20167

[Get15] Getty, P.R., Hardy, L. and Bush, A.M. 2015. Was the Eubrontes track maker gregarious? Testing the herding hypothesis at Powder Hill Dinosaur Park, Middlefield, Connecticut. *Bulletin of the Peabody Museum of Natural History*, 56 (1), pp.95-106. doi: 10.3374/014.056.0109

[Gie91] Gierliński, G.D. 1991. New dinosaur ichnotaxa from the early Jurassic of the Holy cross Mountains, Poland. *Palaeogeography, Palaeoclimatology, Palaeoecology*, 85(1-2), pp.137-148. doi: 10.1016/0031-0182(91)90030-U

[Gie94] Gierliński, G.D and Ahlberg, A., 1994. Late Triassic and Early Jurassic dinosaur footprints in the Höganäs Formation of southern Sweden. *Ichnos*, 3 (2), pp.99-105. doi: 10.1080/10420949409386377

[Gie96] Gierliński, G.D., 1996. Dinosaur ichnotaxa from the Lower Jurassic of Hungary. *Geological Quarterly*, 40 (1), pp.119-128.

[Gie01] Gierliński, G.D., Niedzwiedzki, G. and Pienkowski, G., 2001. Gigantic footprint of a theropod dinosaur in the Early Jurassic of Poland. *Acta Palaeontologica Polonica*, 46 (3). pp.441-446.

[Gie04] Gierliński, G.D., Pieńkowski, G. and Niedźwiedzki, G., 2004. Tetrapod Track Assemblage in the Hettangian of Sołtyków, Poland, and its Paleoenvironmental Background. *Ichnos*, 11 (3-4), pp.195-213. doi: 10.1080/10420940490444861

[Gie08a] Gierliński, G.D., Ploch, I., Gawor-Biedowa, E. and Niedźwiedzki, G. 2008. The first evidence of dinosaur tracks in the Upper Cretaceous of Poland. *Oryctos*, 8, pp.107-113. doi:

[Gie08b] Gierliński, G.D. and Karol, S. 2008. Stegosaurian footprints from the Morrison Formation of Utah and their implications for interpreting other ornithischian tracks. *Oryctos*, 8, pp.29-46.

[Gie09a] Gierliński, G.D., Niedzwiedzki, G. and Nowacki, P. 2009a. Small theropod and ornithopod footprints in the Late Jurassic of Poland. *Acta Geologica Polonica*, 59 (2), pp.221-234. doi:

[Gie09b] Gierliński, G.D., Menducki, P., Janiszewska, K., Wicik, I. and Boczarowski, A. 2009b. A preliminary report on dinosaur track assemblages from the Middle Jurassic of the Imilchil area, Morocco. *Geological Quarterly*, 53 (4), pp.477-482.

[Gie17] Gierliński, G.D., Lagnaoui, A., Klein, K., Saber, H., Oukassou, M. and Charriere, A. 2017. Bird-like tracks from the Imilchil Formation (Middle Jurassic, Bajocian-Bathonian) of the Central High Atlas, Morocco, in comparison with similar Mesozoic tridactylous ichnotaxa. *Bollettino della Società Paleontologica Italiana*, 56 (2), pp.207-215.

[Gon15] González Riga, B.J., David, L.D.O., Tomaselli, M.B., dos Anjos Candeiro, C.R., Coria, J.P. and Prámparo, M. 2015. Sauropod and theropod dinosaur tracks from the Upper Cretaceous of Mendoza (Argentina): trackmakers and anatomical evidences. *Journal of South American Earth Sciences*, *61*, pp.134-141. doi: 10.1016/j.jsames.2014.11.006

[Gui22] Guillaume, A.R.D., Costa, F. and Mateus, O. 2022. Stegosaur tracks from the Upper Jurassic of Portugal: new occurrences and perspectives. *Ciências Da Terra/Earth Sciences Journal*, 20 (1), pp.37-60. doi: 10.21695/cterraesj.v20i1.437

[Had21] Hadland, P.T., Friedrich, S., Lagnaoui, A. and Martill, D.M. 2021. The youngest dinosaur footprints from England and their palaeoenvironmental implications. *Proceedings of the Geologists' Association*, 132 (4), pp.479-490. doi: 10.1016/j.pgeola.2021.04.005

[Hal16] Hall, L.E., Fragomeni, A.E. and Fowler, D.W. 2016. The flexion of sauropod pedal unguals and testing the substrate grip hypothesis using the trackway fossil record. In Falkingham, P.L., Marty, D. and Richter, A. Dinosaur Tracks: The Next Steps. Bloomington: Indiana University Press. 138-151 pp.

[He13] He, Q., Xing, L., Zhang, J., Lockley, M.G., Klein, H., Persons IV, W.S., Qi, L. and Jia, C. 2013. New Early Cretaceous Pterosaur-Bird Track Assemblage from Xinjiang, China:

Palaeoethology and Palaeoenvironment. *Acta Geologica Sinica-English Edition*, 87 (6), pp.1477-1485. doi: 10.1111/1755-6724.12151

[Hen17] Henderson, D.M. 2017. The first evidence of iguanodontids (Dinosauria: Ornithischia) in Alberta, Canada – a fossil footprint from the Early Cretaceous. *Cretaceous Research*, 76, pp.28-33. doi: 10.1016/j.cretres.2017.04.015

[Her22] Herrera-Castillo, C.M., Moratalla, J.J., Belaústegui, Z., Marugán-Lobón, J., Martín-Abad, H., Nebreda, S.M., López-Archilla, A.I. and Buscalioni, A.D. 2022. A theropod trackway providing evidence of a pathological foot from the exceptional locality of Las Hoyas (upper Barremian, Serranía de Cuenca, Spain). *PLoS ONE*, 17 (4), p.e0264406. doi: 10.1371/journal.pone.0264406

[Her16] Herrero, C., Herrero, E., Martín-Chivelet, J. and Pérez-Lorente, F. 2016. Contribution to knowledge of the last dinosaur footprints in Europe. Persistence of ornithopods in the upper Maastrichtian of SE Spain. *Cretaceous Research*, 57, pp.490-507. doi: 10.1016/j.cretres.2015.05.011

[Hor14] Hornung, J.J. and Reich, M. 2014. *Metatetrapous valdensis* Nopcsa, 1923 and the presence of ankylosaur tracks (Dinosauria: Thyreophora) in the Berriasian (Early Cretaceous) of Northwestern Germany. *Ichnos*, *21*(1), pp.1-18. doi: 10.1080/10420940.2013.873720

[Hor16] Hornung, J.J., Böhme, A., Schlüter, N. and Reich, M. 2016. Diversity, ontogeny, or both? A morphometric approach to iguanodontian ornithopod (Dinosauria: ornithischia) track assemblages from the Berriasian (Lower Cretaceous) of North Western Germany. In Falkingham, P.L., Marty, D. and Richter, A. Dinosaur Tracks: The Next Steps. Bloomington: Indiana University Press. 202-225 pp.

[Huh03] Huh, M., Hwang, K.G., Paik, I.S., Chung, C.H. and Kim, B.S. 2003. Dinosaur tracks from the Cretaceous of South Korea: Distribution, occurrences and paleobiological significance. *Island Arc*, 12 (2), pp.132-144. doi: 10.1046/j.1440-1738.2003.00386.x

[Hun98] Hunt, A.P. and Lucas, S. 1998. Tetrapod ichnofaunas from the Lower Cretaceous of northeastern New Mexico, USA. In Lucas, S.G., Kirkland, J.I. and Estep, J.W. (eds.) Lower and Middle Cretaceous Terrestrial Ecosystems. *New Mexico Museum of Natural History and Science Bulletin*, 14, pp.163-167.

[Ima18] Imai, T., Tsukiji, Y. and Azuma, Y. 2018. Description of bird tracks from the Kitadani Formation (Aptian), Katsuyama, Fukui, Japan with three-dimentional imaging techniques. *Memoir of the Fukui Prefectural Dinosaur Museum*, *17*, pp.1-8.

[Ish89] Ishigaki, S. and Fujisaki, T. 1989. Three dimensional representation of Eubrontes by the method of Moiré topography. In Gillette, D.D. and Lockley, M.G. (eds.) Dinosaur Tracks and Traces. Cambridge: Cambridge University Press. 421-425 pp.

[Ish10] Ishigaki, S. 2010. Theropod trampled bedding plane with laboring trackways from the Upper Cretaceous Abdrant Nuru fossil site, Mongolia. *Hayashibara Museum of Natural Sciences Research Bulletin*, 3, pp.133-141.

[Kan21] Kang, S.H., Buckley, L.G., McCrea, R.T., Kim, K.S., Lockley, M.G., Lim, J.D., Lim, H.S. and Kim, C.B. 2021. First report of bird tracks (Ignotornis seoungjoseoi ichnosp. nov.) from the Jinju Formation (Lower Cretaceous), Sacheon City, Korea. Cretaceous Research, 127, p.104899. doi: 10.1016/j.cretres.2021.104899

[Kel12] Kellner, A.W., Dalla Vecchia, F.M., Mirzaie Ataabadi, M., De Paula Silva, H. and Khosravi, E. 2012. Review of the dinosaur record from Iran with the description of new

material. *Rivista Italiana di Paleontologia e Stratigrafia (Research In Paleontology and Stratigraphy)*, 118 (2), pp.261-275.

[Kim06] Kim, J.Y., Kim, S.H., Kim, K.S. and Lockley, M. 2006. The oldest record of webbed bird and pterosaur tracks from South Korea (Cretaceous Haman Formation, Changseon and Sinsu Islands): more evidence of high avian diversity in East Asia. *Cretaceous research*, 27 (1), pp.56-69. doi: 10.1016/j.cretres.2005.10.005

[Kim09] Kim, J.Y., Lockley, M.G., Kim, H.M., Lim, J.D. and Kim, K.S. 2009. New dinosaur tracks from Korea, *Ornithopodichnus masanensis* ichnogen. et ichnosp. nov. (Jindong Formation, Lower Cretaceous): implications for polarities in ornithopod foot morphology. *Cretaceous Research*, 30 (6), pp.1387-1397. doi: 10.1016/j.cretres.2009.08.003

[Kim12a] Kim, J.Y., Lockley, M.G., Seo, S.J., Kim, K.S., Kim, S.H. and Baek, K.S. 2012. A paradise of Mesozoic birds: the world's richest and most diverse Cretaceous bird track assemblage from the Early Cretaceous Haman Formation of the Gajin tracksite, Jinju, Korea. *Ichnos*, 19(1-2), pp.28-42. doi: 10.1080/10420940.2012.660414

[Kim12b] Kim, J.Y. and Lockley, M.G., 2012. New sauropod tracks (*Brontopodus pentadactylus* ichnosp. nov.) from the Early Cretaceous Haman Formation of Jinju Area, Korea: implications for sauropods manus morphology. *Ichnos*, 19(1-2), pp.84-92. doi: 10.1080/10420940.2012.664056

[Kim13] Kim, J.Y., Kim, M.K., Oh, M.S. and Lee, C.Z. 2013. A new semi-palmate bird track, *Gyeongsangornipes lockleyi* ichnogen. et ichnosp. nov., and *Koreanaornis* from the Early Cretaceous Jindong Formation of Goseong County, southern coast of Korea. *Ichnos*, 20(2), pp.72-80. doi: 10.1080/10420940.2013.787071

[Kim16] Kim, J.Y., Lockley, M.G and Chun, H.Y. 2016. New dinosaur tracks from the Lower Cretaceous (Valanginian-Hauterivian) Saniri Formation of Yeongdong area, central Korea: Implications for quadrupedal ornithopod locomotion. *Cretaceous Research*, 61, pp.5-16. doi: 10.1016/j.cretres.2015.12.023

[Kim17] Kim, K.S., Lim, J.D., Lockley, M.G., Xing, L., Ha, S.J., Kim, C.B., Paik, I.S., Ahn, J.H. and Mun, S.C. 2017. First reports of a distinctive theropod track assemblage from the Jinju Formation (Lower Cretaceous) of Korea provides strong correlations with China. *Cretaceous Research*, 81, pp.26-35. doi: 10.1016/j.cretres.2017.08.005

[Kle20] Klein, H., Gierliński, G., Lallensack, J.N., Abu Hamad, A., Al-Mashakbeh, H., Alhejoj, I., Konopka, M. and Błoński, M. 2020. First Upper Cretaceous dinosaur track assemblage from Jordan (Middle East): preliminary results. *Annales Societatis Geologorum Poloniae*, 90, 331–342, doi: 10.14241/asgp.2020.10.

[Kle23] Klein, H., Gierliński, G.D., Oukassou, M., Saber, H., Lallensack, J.N., Lagnaoui, A., Hminna, A. and Charriere, A. 2023. Theropod and ornithischian dinosaur track assemblages from Middle to? Late Jurassic deposits of the Central High Atlas, Morocco. *Historical Biology*, 35 (3), pp.320-346. doi: 10.1080/08912963.2022.2042808

[Kub89] Kuban, G.J. 1989. Elongate dinosaur tracks. In Gillette, D.D. and Lockley, G.M. (eds.) Dinosaur Tracks and Traces. Cambridge: Cambridge University Press. 57–72 pp.

[Lal15] Lallensack, J.N., Sander, M., Knötschke, N. and Wings, O., 2015. Dinosaur tracks from the Langenberg Quarry (Late Jurassic, Germany) reconstructed with historical photogrammetry: evidence for large theropods soon after insular dwarfism. *Palaeontologia Electronica*, 18.2.31A: pp.1-34. doi: 10.26879/529

[Lal16] Lallensack, J.N., van Heteren, A.H. and Wings, O., 2016. Geometric morphometric analysis of intratrackway variability: a case study on theropod and ornithopod dinosaur trackways from Münchehagen (Lower Cretaceous, Germany). *PeerJ*, *4*, p.e2059. doi: 10.7717/peerj.2059

[Lal22a] Lallensack, J.N., Bordy, E.M., Lockley, M.G. and Wings, O. 2022a. Relaunching the TY tracksite: tridactyl dinosaur footprints from the Lower Jurassic of southern Africa. *Historical Biology*, 35 (10), pp.1782-1793. doi: 10.1080/08912963.2022.2117042

[Lal22b] Lallensack, J.N., Romilio, A. and Falkingham, P.L., 2022b. A machine learning approach for the discrimination of theropod and ornithischian dinosaur tracks. Journal of the Royal Society Interface, 19(196), p.20220588. doi: 10.1098/rsif.2022.0588

[Lal22c] Lallensack, J.N., Farlow, J.O. and Falkingham, P.L., 2022c. A new solution to an old riddle: elongate dinosaur tracks explained as deep penetration of the foot, not plantigrade locomotion. *Palaeontology*, *65*(1), p.e12584. doi: 10.1111/pala.12584

[Lal22d] Lallensack, J.N. and Wings, O. 2022. 3D model and photographs of a theropod dinosaur track from Munchehagen (2015). Figshare. Available at: https://doi.org/10.6084/m9.figshare.20395419.v1

[LeL99] Le Lœuff, J., Lockley, M.G., Meyer, C. and Petit, J.P., 1999. Discovery of a thyreophoran trackway in the Hettangian of central France. *Comptes Rendus de l'Académie des Sciences-Series IIA-Earth and Planetary Science*, 328 (3), pp.215-219. doi: 10.1016/S1251-8050(99)80099-8

[Lee18] Lee, Y.N., Lee, H.J., Han, S.Y., Park, E. and Lee, C.H., 2018. A new dinosaur tracksite from the Lower Cretaceous Sanbukdong Formation of Gunsan City, South Korea. *Cretaceous Research*, *91*, pp.208-216. doi: 10.1016/j.cretres.2018.06.003

[Leo80] Leonardi, G. 1980. Dez novas pistas de dinossauros (Theropoda Marsh, 1881) na bacia do Rio do Peixe, Paraíba, Brasil. *Actas II Congreso Argentino de Paleontología y Bioestratigrafía y I Congreso Latinoamericano de Paleontología*, 1.

[Leo04] Leonardi, G. and Santos, M.D.F.C.F.D. 2004. New dinosaur tracksites from the Sousa Lower Cretaceous basin (Paraíba, Brasil). *Studi trentini di scienze naturali - Acta Geologica*, 81, pp.5–21.

[Leo21a] Leonardi, G. and and de Souza Carvalho, I. 2021. Dinosaur Tracks from Brazil: A Lost World of Gondwana. Bloomington: Indiana University Press.

[Leo21b] Leonardi, G., Santos, M.D.F.C.F.D. and Barbosa, F.H.D.S. 2021. First dinosaur tracks from the Açu Formation, Potiguar Basin (mid-Cretaceous of Brazil). *Anais da Academia Brasileira de Ciências*, 93 (2), p.e20210635. doi: 10.1590/0001-3765202120210635

[Li21] Li, H., de Fabrègues, C.P., Bi, S., Wang, Y. and Xu, X. 2021. The largest theropod track site in Yunnan, China: a footprint assemblage from the Lower Jurassic Fengjiahe Formation. *PeerJ*, *9*, p.e11788. doi: 10.7717/peerj.11788

[Li12] Li, J., Lockley, M.G., Yuguang, Z., Songmei, H., Masaki, M. and Zhiqiang, B. 2012. An important ornithischian tracksite in the Early Jurassic of the Shenmu Region, Shaanxi, China. *Acta Geologica Sinica-English Edition*, 86 (1), pp.1-10. doi: 10.1111/j.1755-6724.2012.00606.x

[Li05] Li, R., Lockley, M.G. and Liu, M., 2005. A new ichnotaxon of fossil bird track from the early cretaceous Tianjialou formation (Barremian-Albian), Shandong province, China. Chinese Science Bulletin, 50 (11), pp.1149-1154. doi: 10.1360/982004-823

[Li11] Li, R., Lockley, M.G., Matsukawa, M., Wang, K. and Liu, M., 2011. An unusual theropod track assemblage from the Cretaceous of the Zhucheng area, Shandong Province, China. *Cretaceous Research*, *32*(4), pp.422-432.

[Li15] Li, R., Lockley, M.G., Matsukawa, M. and Liu, M. 2015. Important Dinosaur-dominated footprint assemblages from the Lower Cretaceous Tianjialou Formation at the Houzuoshan Dinosaur Park, Junan County, Shandong Province, China. *Cretaceous Research*, *52*, pp.83-100. doi: 10.1016/j.cretres.2014.08.004

[Li23] Li, Y., Yao, H., Yu, Y., Foster, W.J., Wang, C., Zhao, L. and Xing, L. 2023. First report of sauropod and ornithopod tracks from the Upper Cretaceous of Tibet, China. *Cretaceous Research*, *149*, p.105569. doi: 10.1016/j.cretres.2023.105569

[Lim12] Lim, J.D., Lockley, M.G. and Kong, D.Y., 2012. The trackway of a quadrupedal ornithopod from the Jindong Formation (Cretaceous) of Korea. *Ichnos*, *19*(1-2), pp.101-104. doi: 10.1080/10420940.2012.664059

[Lin23] Linares Montes, M., Luzón, A., Cuenca-Bescos, G., Canudo, J.I. and Castanera, D., 2023. New mammal and bird tracks from the Lower Oligocene of the Ebro Basin (NE Spain): implications for the Palaeogene ichnological record. Historical Biology, 35(9), pp.1616-1636. doi: 10.1080/08912963.2022.2104644

[Lin03] Lingham-Soliar, T., Broderick, T. and Ait Kaci Ahmed, A. 2003. Closely associated theropod trackways from the Jurassic of Zimbabwe. *Naturwissenschaften*, 90, pp.572-576. doi: 10.1007/s00114-003-0477-5

[Loc87] Lockley, M.G. 1987. Dinosaur footprints from the Dakota Group of Eastern Colorado. *The Mountain Geologist*, 24, pp.107-122.

[Loc94a] Lockley, M.G. and Hunt, A.P. 1994. A track of the giant theropod dinosaur Tyrannosaurus from close to the Cretaceous/Tertiary boundary, northern New Mexico. *Ichnos*, 3, pp.213-218. doi: 10.1080/10420949409386390

[Loc94b] Lockley, M.G., Farlow, J.O. and Meyer, C.A., 1994. *Brontopodus* and *Parabrontopodus* ichnogen. nov. and the significance of wide-and narrow-gauge sauropod trackways. *Gaia*, *10*, pp.135-145.

[Loc95] Lockley, M.G. and Hunt, A.P. 1995. Dinosaur tracks and other fossil footprints of the western United States. New York: Columbia University Press.

[Loc96] Lockley, M.G., King, M., Howe, S. and Sharp, T. 1996. Dinosaur tracks and other archosaur footprints from the Triassic of South Wales. Ichnos: An International Journal of Plant & Animal, 5 (1), pp.23-41. doi: 10.1080/10420949609386404

[Loc98a] Lockley, M.G. and Matsukawa, M. 1998. Lower Cretaceous vertebrate tracksites of east Asia. In Lucas, S.G., Kirkland, J.I. and Estep, J.W. (eds.) Lower and Middle Cretaceous Terrestrial Ecosystems. *New Mexico Museum of Natural History and Science Bulletin*, 14, pp. 135–142.

[Loc98b] Lockley, M.G., Meyer, C. and Santos, V.F.D. 1998b. Megalosauripus and the problematic concept of megalosaur footprints. *Gaia*, *15*, pp.313-337.

[Loc98c] Lockley, M.G., Meyer, C.A. and Moratalla, J.J. 1998c. Therangospodus: trackway evidence for the widespread distribution of a Late Jurassic theropod with well-padded feet. *Gaia*, 15, pp.339–353.

[Loc98d] Lockley, M.G., Hunt, A.P., Meyer, C., Rainforth, E.C. and Schultz, R.J., 1998d. A survey of fossil footprint sites at Glen Canyon National Recreation Area (western USA): a case study in documentation of trace fossil resources at a national preserve. *Ichnos*, 5 (3), pp.177-211. doi: 10.1080/10420949809386417

[Loc98e] Lockley, M.G., Meyer, C., Siber, H.-J. and Pabst, B. 1998e. Theropod tracks from the Howe Quarry, Morrison Formation, Wyoming. *Modern Geology*, 23, pp.309-316.

[Loc00a] Lockley, M.G., Lucas, S.G. and Hunt, A.P. 2000. Dinosaur tracksites in New Mexico: a review. In Lucas, S.G. and Heckert, A.B. (eds.) Dinosaurs of New Mexico. *New Mexico Museum of Natural History Bulletin*, 17, pp.9-16.

[Loc00b] Lockley, M.G. and Meyer, C. 2000. Dinosaur tracks and other fossil footprints of Europe. New York: Columbia University Press.

[Loc01] Lockley, M.G., Janke, P. and Theisen, L. 2001. First reports of bird and ornithopod tracks from the Lakota Formation (Early Cretaceous), Black Hills, South Dakota. In Tanke, D.H., Carpenter, K. and Skrepnick, M.W. (eds.) Mesozoic Vertebrate Life: New Research Inspired by the Paleontology of Philip J. Currie. Bloomington: Indiana University Press. 443–452 pp.

[Loc02] Lockley, M.G. and Rainforth, E.C. 2002. The track record of Mesozoic birds and pterosaurs. In Chiappe, L.M. and Witmer, L.M. (eds.) Mesozoic Birds: Above the Heads of Dinosaurs. Berkeley: University of California Press. 405–418 pp.

[Loc03] Lockley, M.G., Nadon, G. and Currie, P.J. 2003. A diverse dinosaur-bird footprint assemblage from the Lance Formation, Upper Cretaceous, Eastern Wyoming: implications for ichnotaxonomy. *Ichnos*, 11, pp. 229-249. doi: 10.1080/10420940490428625

[Loc04] Lockley, M.G., Wright, J.L. and Thies, D., 2004. Some observations on the dinosaur tracks at Münchehagen (Lower Cretaceous), Germany. *Ichnos*, 11 (3-4), pp.261-274. doi: 10.1080/10420940490428805

[Loc06a] Lockley, M.G. and Gierliński, G.D. 2006a. Diverse vertebrate ichnofaunas containing Anomoepus and other unusual trace fossils from the Lower Jurassic of the western United States: implications for paleoecology and palichnostratigraphy. In Harris, J.D., Lucas, S.G., Spielmann, J.A., Lockley, M.G., Milner, A.R.C. and Kirkland, J.I. (eds.) The Triassic-Jurassic Terrestrial Transition. *New Mexico Museum of Natural History and Science Bulletin*, 37, pp.176-191.

[Loc06b] Lockley, M.G., Gierliński, G.D., Titus, A.L. and Albright, B. 2006b. An introduction to thunderbird footprints at the Flag Point pictograph-track site-preliminary observations on Lower Jurassic theropod tracks from the Vermillion Cliffs area, southwestern Utah. In Harris, J.D., Lucas, S.G., Spielmann, J.A., Lockley, M.G., Milner, A.R.C. and Kirkland, J.I. (eds.) The Triassic-Jurassic Terrestrial Transition. *New Mexico Museum of Natural History and Science Bulletin*, 37, 310–314 pp.

[Loc06c] Lockley, M.G., Matsukawa, M., Sato, Y., Polahan, M. and Daorerk, V. 2006c. A distinctive new theropod dinosaur track from the Cretaceous of Thailand: implications for theropod track diversity. *Cretaceous Research*, 27 (1), pp.139-145. doi: 10.1016/j.cretres.2005.10.002

[Loc06d] Lockley, M.G., Matsukawa, M. and Witt, D. 2006d. Giant theropod tracks from the Cretaceous Dakota group of Northeastern New Mexico. In Lucas, S.G. and Sullivan, R.M. (eds.) Late Cretaceous Vertebrates of the Western Interior. *New Mexico Museum of Natural History and Science Bulletin*, 35, pp.83-87.

[Loc06e] Lockley, M.G., Kukihara, R. and Mitchell, L. 2006e. New dinosaur and crocodile tracksites from the Cretaceous Dakota Group of the Colorado front range, Boulder and Jefferson Counties, Colorado. In Lucas, S.G. and Sullivan, R.M. (eds.) Late Cretaceous Vertebrates of the Western Interior. *New Mexico Museum of Natural History and Science Bulletin*, 35, pp.89-94.

[Loc06f] Lockley, M.G., Holbrook, J., Kukihara, R. and Matsukawa, M., 2006f. An ankylosaur-dominated dinosaur tracksite in the Cretaceous Dakota Group of Colorado: paleoenvironmental and sequence stratigraphic context. In Lucas, S.G. and Sullivan, R.M. (eds.) Late Cretaceous Vertebrates from the Western Interior. *New Mexico Museum of Natural History and Science Bulletin*, *35*, pp.95-104.

[Loc07a] Lockley, M.G., Mitchell, L. and Odier, G.P. 2007a. Small theropod track assemblages from Middle Jurassic eolianites of Eastern Utah: paleoecological insights from dune ichnofacies in a transgressive sequence. *Ichnos*, 14 (1-2), pp.131-142. doi: 10.1080/10420940601010901

[Loc07b] Lockley, M.G., Li, R., Harris, J.D., Matsukawa, M. and Liu, M., 2007b. Earliest zygodactyl bird feet: evidence from Early Cretaceous roadrunner-like tracks. *Naturwissenschaften*, 94 (8), pp.657-665. doi: 10.1007/s00114-007-0239-x

[Loc08a] Lockley, M.G., Garcia-Ramos, J.C., Pinuela, L. and Avanzini, M. 2008a. A review of vertebrate track assemblages from the Late Jurassic of Asturias, Spain with comparative notes on coeval ichnofaunas from the western USA: implications for faunal diversity in siliciclastic facies assemblages. *Oryctos*, 8, pp.53-70.

[Loc08b] Lockley, M.G., Kim, J.Y., Kim, K.S., Kim, S.H., Matsukawa, M., Rihui, L., Jianjun, L. and Yang, S.Y. 2008b. Minisauripus—the track of a diminutive dinosaur from the Cretaceous of China and South Korea: implications for stratigraphic correlation and theropod foot morphodynamics. *Cretaceous Research*, 29 (1), pp.115-130. doi: 10.1016/j.cretres.2007.04.003

[Loc09] Lockley, M.G., McCrea, R.T. and Matsukawa, M., 2009. Ichnological evidence for small quadrupedal ornithischians from the basal Cretaceous of SE Asia and North America: implications for a global radiation. *Geological Society, London, Special Publications*, 315, pp. 255-269.

[Loc11] Lockley, M.G., Cart, K., Martin, J. and Milner, A.R.C. 2011. New theropod tracksites from the Upper Cretaceous "Mesaverde" Group, western Colorado: implications for ornithomimosaur track morphology. In Sullivan, R.M., Lucas, S.G. and Spielmann, J.A. (eds.) Fossil Record 3. *New Mexico Museum of Natural History and Science Bulletin*, 53, pp.321-329.

[Loc12] Lockley, M.G., Huh, M. and Kim, B.S. 2012. *Ornithopodichnus* and pes-only sauropod trackways from the Hwasun tracksite, Cretaceous of Korea. *Ichnos*, 19 (1-2), pp.93-100. doi: 10.1080/10420940.2011.625726

[Loc13] Lockley, M.G., Li, J., Li, R., Matsukawa, M., Harris, J.D. and Lida, X. 2013. A review of the tetrapod track record in China, with special reference to type ichnospecies: implications for ichnotaxonomy and paleobiology. *Acta Geologica Sinica‐English Edition*, 87 (1), pp.1-20. doi: 10.1111/1755-6724.12026

[Loc14] Lockley, M.G. and Gierliński, G.D., 2014. Notes on a new ankylosaur track from the Dakota Group (Cretaceous) of Northern Colorado. In Lockley, M.G. and Lucas, S.G. (eds.) Fossil footprints of western North America. *New Mexico Museum of Natural History and Science Bulletin*, 62, pp.301-306.

[Loc14a] Lockley, M.G., Gierliński, G.D., Houck, K., Lim, J.D., Kim, K.S., Kim, D.Y., Kim, T.K., Kang, S.H., Hunt-Foster, R., Li, R. and Chesser, C., 2014a. New excavations at the Mill Canyon

dinosaur track site (Cedar Mountain Formation, Lower Cretaceous) of eastern Utah. In Lockley, M.G. and Lucas, S.G. (eds.) Fossil footprints of western North America. *New Mexico Museum of Natural History and Science Bulletin*, 62, pp.287-300.

[Loc14b] Lockley, M.G., Gierliński, G.D., Martin, J. and Kent, C. 2014b. An unusual theropod tracksite in the Cretaceous Dakota Group, western Colorado: implications for ichnodiversity. In Lockley, M.G. and Lucas, S.G. (eds.) Fossil footprints of western North America. *New Mexico Museum of Natural History and Science Bulletin*, 62, pp.411–415.

[Loc14c] Lockley, M.G., Foster, R.H., Foster, J., Cart, K. and Gerwe, S., 2014c. Early Jurassic track assemblages from the Granite Creek Area of Eastern Utah. In Lockley, M.G. and Lucas, S.G. (eds.) Fossil footprints of western North America. *New Mexico Museum of Natural History and Science Bulletin*, 62, pp.205-210.

[Loc14d] Lockley, M.G., Gierliński, G.D., Dubicka, Z., Breithaupt, B.H., Matthews, N.A. 2014d. A preliminary report on a new dinosaur tracksite in the Cedar Mountain Formation (Cretaceous) of Eastern Utah. In Lockley, M.G. and Lucas, S.G. (eds.) Fossil footprints of western North America. *New Mexico Museum of Natural History and Science Bulletin*, 62, pp.279–285.

[Loc14e] Lockley, M.G., Honda, K.K. and Simmons, B. 2014e. A new dinosaur tracksite in the Dakota Group (Cretaceous) of the historic Stone City area, Colorado. In Lockley, M.G. and Lucas, S.G. (eds.) Fossil footprints of western North America. *New Mexico Museum of Natural History and Science Bulletin*, 62, pp.355-359.

[Loc14f] Lockley, M.G., Houck, K., Green, C. and Caldwell, M. 2014f. New fossil footprints from the Dakota Group (Cretaceous) Roxborough State Park, Colorado. In Lockley, M.G. and Lucas, S.G. (eds.) Fossil footprints of western North America. *New Mexico Museum of Natural History and Science Bulletin*, 62, pp.373–384.

[Loc14g] Lockley, M.G., Cart, K., Martin, J., Prunty, R., Houck, K., Hups, K., Lim, J.D., Kim, K.S. and Gierliński, G. 2014g. A bonanza of new tetrapod tracksites from the Cretaceous Dakota Group, Western Colorado: Implications for paleoecology. In Lockley, M.G. and Lucas, S.G. (eds.) Fossil footprints of western North America. *New Mexico Museum of Natural History and Science Bulletin*, 62, pp.393–409.

[Loc14h] Lockley, M.G., Triebold, M. and Janke, P.R. 2014h. Dinosaur tracks from the Hell Creek Formation (Upper Cretaceous, Maastrichtian), South Dakota. In Lockley, M.G. and Lucas, S.G. (eds.) Fossil footprints of western North America. *New Mexico Museum of Natural History and Science Bulletin*, 62, pp.459–468.

[Loc14i] Lockley, M.G., Xing, L., Lockwood, J.A. and Pond, S. 2014i. A review of large Cretaceous ornithopod tracks, with special reference to their ichnotaxonomy. *Biological Journal of the Linnean Society*, 113 (3), pp.721-736. doi: 10.1111/bij.12294

[Loc15a] Lockley, M.G. and Xing, L. 2015. Flattened fossil footprints: implications for paleobiology. *Palaeogeography, Palaeoclimatology, Palaeoecology*, *426*, pp.85-94. doi: 10.1016/j.palaeo.2015.03.008

[Loc15b] Lockley, M.G., Buckley, L.G., Foster, J.R., Kirkland, J.I. and DeBlieux, D.D., 2015. First report of bird tracks (Aquatilavipes) from the Cedar Mountain Formation (Lower Cretaceous), eastern Utah. *Palaeogeography, Palaeoclimatology, Palaeoecology*, *420*, pp.150-162. doi: 10.1016/j.palaeo.2014.12.014

[Loc18a] Lockley, M.G., Burton, R. and Grondel, L. 2018a. A large assemblage of tetrapod tracks from the Cretaceous Naturita Formation, Cedar Canyon region, southwestern Utah. *Cretaceous Research*, *92*, pp.108-121. doi: 10.1016/j.cretres.2018.08.003

[Loc18b] Lockley, M.G., Foster, J.R. and Foster, R.H. 2018b. The first North American *Deltapodus* trackway in a diverse *Anomoepus*, theropod, sauropod, and turtle track assemblage from the Upper Jurassic Salt Wash Member (Morrison Formation) of eastern Utah. In Lucas, S.G. and Sullivan, R.M. (eds.) Fossil Record 6. *New Mexico Museum of Natural History and Science Bulletin*, 79, pp.407-415.

[Loc18c] Lockley, M.G., Li, J., Xing, L., Guo, B. and Matsukawa, M., 2018c. Large theropod and small sauropod trackmakers from the lower cretaceous jingchuan formation, Inner Mongolia, China. *Cretaceous Research*, *92*, pp.150-167. doi: 10.1016/j.cretres.2018.07.007

[Loc18d] Lockley, M.G. 2018d. A large assemblage of dinosaur tracks from a valley-fill deposit in the Dakota Sandstone, Central Utah: the Cretaceous fossil footprint database expands westward. In Lucas, S.G. and Sullivan, R.M. (eds.) Fossil Record 6. *New Mexico Museum of Natural History and Science Bulletin*, 79, pp.375–385.

[Loc18e] Lockley, M.G. and Doran, B. 2018e. Forgotten footprints: dinosaur tracks and other traces from the High Country of Colorado. In Lucas, S.G. and Sullivan, R.M. (eds.) Fossil Record 6. *New Mexico Museum of Natural History and Science Bulletin*, 79, pp.387–394.

[Loc18f] Lockley, M.G., Matthews, N. A., Breithaupt, B. H., Gierliński, G., Cart, K. and Foster, R.H. 2018f. Large Dinosaur Tracksites in the Lower Jurassic Kayenta Formation near Moab, Utah: implications for Paleoecology. In Lucas, S.G. and Sullivan, R.M. (eds.) Fossil Record 6. *New Mexico Museum of Natural History and Science Bulletin*, 79, pp.441–449.

[Loc18g] Lockley, M.G., Gierliński, G., Adach, L., Schumacher, B. and Cart, K. 2018g. Newly discovered tetrapod ichnotaxa from the Upper Cretaceous Blackhawk Formation, Utah. In Lucas, S.G. and Sullivan, R.M. (eds.) Fossil Record 6. *New Mexico Museum of Natural History and Science Bulletin*, 79, pp.469–480.

[Loc21a] Lockley, M.G., Klein, H., McHugh, J.B. and Romilio, A. 2021a. Fruita's first fossil footprint exhibit: the discovery of forgotten specimens in an historic former museum building. In Lucas, S.G., Hunt, A.P. and Lichtig, A.J. Fossil Record 7. *New Mexico Museum of Natural History and Science Bulletin*, 82, pp.219-226.

[Loc21b] Lockley, M.G., Abbassi, N. and Helm, C.W. 2021b. Large, unwebbed bird and bird-like footprints from the Mesozoic and Cenozoic: a review of ichnotaxonomy and trackmaker affinity. *Lethaia*, *54*(5), pp.969-987. doi: 10.1111/let.12458

[Loc21c] Lockley, M.G., Kim, K.S., Lim, J.D. and Romilio, A. 2021c. Bird tracks from the Green River Formation (Eocene) of Utah: ichnotaxonomy, diversity, community structure and convergence. *Historical Biology*, *33*(10), pp.2085-2102. doi: 10.1080/08912963.2020.1771559

[Lü06] Lü, J.C., Azuma, Y., Wang, T., Li, S.X. and Pan, S.G. 2006. The first discovery of dinosaur footprint from Lufeng of Yunnan Province, China. *Memoir of the Fukui Prefectural Dinosaur Museum*, 5, pp.35-39.

[Luc16] Lucas, S.G. and Dalman, S.G. 2016. The Early Cretaceous Clayton Lake dinosaur tracksite, northeastern New Mexico. In Sullivan, R.M. and Lucas, S.G. (eds.) Fossil Record 5. *New Mexico Museum of Natural History and Science Bulletin*, 74, pp.127-140.

[Lul53] Lull, R.S. 1953. The Triassic life of the Connecticut Valley. *Connecticut State Geological and Natural History Survey Bulletin*, 81, pp.1-331.

[Mar23] Martin, A.J., Lowery, M., Hall, M., Vickers-Rich, P., Rich, T.H., Serrano-Brañas, C.I. and Swinkels, P. 2023. Earliest known Gondwanan bird tracks: Wonthaggi Formation (Early

Cretaceous), Victoria, Australia. *Plos one*, *18*(11), p.e0293308. doi: 10.1371/journal.pone.0293308

[Mar17] Marty, D., Belvedere, M., Razzolini, N.L., Lockley, M.G., Paratte, G., Cattin, M., Lovis, C. and Meyer, C.A. 2017. The tracks of giant theropods (Jurabrontes curtedulensis ichnogen. & ichnosp. nov.) from the Late Jurassic of NW Switzerland: palaeoecological & palaeogeographical implications. *Historical Biology*, 30 (7), pp.928-956. doi: 10.1080/08912963.2017.1324438

[Mar10] Marty, D., Belvedere, M., Meyer, C.A., Mietto, P., Paratte, G., Lovis, C. and Thuering, B. 2010. Comparative analysis of Late Jurassic sauropod trackways from the Jura Mountains (NW Switzerland) and the central High Atlas Mountains (Morocco): implications for sauropod ichnotaxonomy. *Historical Biology*, 22 (1-3), pp.109-133. doi: 10.1080/08912960903503345

[Mat03] Mateus, O. and Antunes, M.T. 2003. A new dinosaur tracksite in the Lower Cretaceous of Portugal. *Ciências da Terra (UNL)*, 15, pp.253.262.

[Mat08] Mateus, O. and Milàn, J. 2008. Ichnological evidence for giant ornithopod dinosaurs in the Upper Jurassic Lourinhã Formation, Portugal. *Oryctos*, 8, pp.47-52.

[Mat10] Mateus, O. and Milàn, J. 2010. A diverse Upper Jurassic dinosaur ichnofauna from central-west Portugal. *Lethaia*, 43 (2), pp.245-257. doi: 10.1111/j.1502-3931.2009.00190.x

[Mat11] Mateus, O., Milàn, J., Romano, M. and Whyte, M.A. 2011. New finds of stegosaur tracks from the Upper Jurassic Lourinhã Formation, Portugal. *Acta Palaeontologica Polonica*, *56*(3), pp.651-658. doi: 10.4202/app.2009.0055

[Mat97] Matsukawa, M., Hamuro, T., Mizukami, T. and Fujii, S. 1997. First trackway evidence of gregarious dinosaurs from the Lower Cretaceous Tetori Group of eastern Toyama Prefecture, central Japan. *Cretaceous Research*, *18*(4), pp.603-619. doi: 10.1006/cres.1997.0075

[Mat05] Matsukawa, M., Shibata, K., Kukihara, R., Koarai, K. and Lockley, M.G., 2005. Review of Japanese dinosaur track localities: implications for ichnotaxonomy, paleogeography and stratigraphic correlation. *Ichnos*, *12*(3), pp.201-222. doi: 10.1080/10420940591009231

[Mat06] Matsukawa, M., Lockley, M. and Jianjun, L. 2006. Cretaceous terrestrial biotas of East Asia, with special reference to dinosaur-dominated ichnofaunas: towards a synthesis. *Cretaceous Research*, 27 (1), pp.3-21. doi: 10.1016/j.cretres.2005.10.009

[McC14a] McCrea, R.T., Buckley, L.G., Farlow, J.O., Lockley, M.G., Currie, P.J., Matthews, N.A. and Pemberton, S.G., 2014a. A 'terror of tyrannosaurs': the first trackways of tyrannosaurids and evidence of gregariousness and pathology in Tyrannosauridae. *PLoS One*, 9 (7), p.e103613. doi: 10.1371/journal.pone.0103613

[McC14b] McCrea, R.T., Buckley, L.G., Plint, A.G., Currie, P.J., Haggart, J.W., Helm, C.W. and Pemberton, S.G., 2014b. A review of vertebrate track-bearing formations from the Mesozoic and earliest Cenozoic of western Canada with a description of a new theropod ichnospecies and reassignment of an avian ichnogenus. In In Lockley, M.G. and Lucas, S.G. (eds.) Fossil footprints of western North America. *New Mexico Museum of Natural History and Science Bulletin*, 62, pp.5-93.

[McC14c] McCrea, R.T. and Pigeon, T.S. 2014c. Replication and description of a large theropod and large ornithopod trackway from the upper Minnes Group (Lower Cretaceous: Valanginian) of the Peace Region of northeastern British Columbia, Canada. In Lockley, M.G. and Lucas, S.G. (eds.) Fossil footprints of western North America. *New Mexico Museum of Natural History and Science Bulletin*, 62, pp.269-277.

[Mey03] Meyer, C.A. and Thuring, B. 2003. The first iguanodontid dinosaur tracks from the Swiss Alps (Schrattenkalk Formation, Aptian). *Ichnos*, 10 (2-4), pp.221-228. doi: 10.1080/10420940390256186

[Mey21] Meyer, C.A., Belvedere, M., Englich, B. and Lockley, M.G., 2021. A reevaluation of the Late Jurassic dinosaur tracksite Barkhausen (Wiehengebirge, Northern Germany). *PalZ*, *95*(3), pp.537-558.

[Mil15] Milàn, J. and Surlyk, F. 2015. An enigmatic, diminutive theropod footprint in the shallow marine Pliensbachian Hasle Formation, Bornholm, Denmark. *Lethaia*, 48 (4), pp.429-435. doi: 10.1111/let.12115

[Mil09] Milàn, J. and Chiappe, L.M. 2009. First American record of the Jurassic ichnospecies *Deltapodus brodricki* and a review of the fossil record of stegosaurian footprints. *The Journal of Geology*, *117*(3), pp.343-348. doi: 10.1086/597363

[Mil06a] Milner, A.R.C., Vice, G.S., Harris, J.D. and Lockley, M.G. 2006a. Dinosaur tracks from the Upper Cretaceous Iron Springs Formation, Iron County, Utah. In Lucas, S.G. and Sullivan, R.M. Late Cretaceous vertebrates from the Western Interior. *New Mexico Museum of Natural History and Science Bulletin*, 35, pp.105-113.

[Mil06b] Milner, A.R.C., Lockley, M.G. and Johnson, S.B. 2006b. The story of the St. George Dinosaur Discovery Site at Johnson Farm: an important new Lower Jurassic dinosaur tracksite from the Moenave Formation of southwestern Utah. In Harris, J.D., Lucas, S.G., Spielmann, J.A., Lockley, M.G., Milner, A.R.C. and Kirkland, J.I. (eds.) The Triassic-Jurassic Terrestrial Transition. *New Mexico Museum of Natural History and Science Bulletin*, 37, pp.329-345.

[Mor92] Moratalla, J.J., Sanz, J.L., Jiménez, S. and Lockley, M.G. 1992. A quadrupedal ornithopod trackway from the Lower Cretaceous of La Rioja (Spain): inferences on gait and hand structure. *Journal of Vertebrate Paleontology*, 12 (2), pp.150-157. doi: 10.1080/02724634.1992.10011445

[Mor94] Moratalla, J.J., Sanz, J.L. and Jiménez, S. 1994. Dinosaur tracks from the Lower Cretaceous of Regumiel de la Sierra (province of Burgos, Spain): inferences on a new quadrupedal ornithopod trackway. *Ichnos*, 3 (2), pp.89-97. doi: 10.1080/10420949409386376

[Mor19] Moreau, J.D., Trincal, V., Fara, E., Baret, L., Jacquet, A., Barbini, C., Flament, R., Wienin, M., Bourel, B. and Jean, A. 2019. Middle Jurassic tracks of sauropod dinosaurs in a deep karst cave in France. *Journal of Vertebrate Paleontology*, 39 (6), p.e1728286. doi: 10.1080/02724634.2019.1728286

[Mor21] Moreau, J.D., Sciau, J., Gand, G. and Fara, E. 2021. Uncommon preservation of dinosaur footprints in a tidal breccia: Eubrontes giganteus from the Early Jurassic Mongisty tracksite of Aveyron, southern France. *Geological Magazine*, 158 (8), pp.1403-1420. doi: 10.1017/S0016756820001454

[Mus12] Mustoe, G.E., Tucker, D.S. and Kemplin, K.L. 2012. Giant Eocene bird footprints from northwest Washington, USA. *Palaeontology*, 55 (6), pp.1293-1305. doi: 10.1111/j.1475-4983.2012.01195.x

[Net23] Neto de Carvalho, C., Belo, J., Figueiredo, S., Cunha, P.P., Muniz, F., Belaustegui, Z., Cachão, M., Rodriguez-Vidal, J., Caceres, L.M., Baucon, A. and Murray, A.S. 2023. Coastal raptors and raiders: New bird tracks in the Pleistocene of SW iberian Peninsula. *Quaternary Science Reviews*, *313*, p.108185. doi: 10.1016/j.quascirev.2023.108185

[Nie04] Niedźwiedzki, G. and Pieńkowski, G. 2004. A dinosaur track association from the Early Jurassic deltaic deposits of Podole near Opatów, Poland. *Geological Quarterly*, 48 (4), pp.333-338. doi:

[Nie16] Niedziedzki, G. and Pienkowski, G. 2016. A dinosaur track assemblage from the Upper Hettangian (Lower Jurassic) marginal-marine deposits of Zapniow, Holy Cross Mountains, Poland. *Geological Quarterly*, 60 (4), pp.840-856.

[Niu23] Niu, K. and Xing, L. 2023. The first dinosaur track assemblages from the Upper Cretaceous Shaxian Formation, Fujian Province, southeastern China. *Cretaceous Research*, 146, p.105486. doi: 10.1016/j.cretres.2023.105486

[Ols80] Olsen, P.E. 1980 Fossil great lakes of the Newark Supergroup in New Jersey. *Field Studies in New Jersey Geology and Guide to Field Trips, 52nd Annual Meeting of New York State Geological Association, Newark College of Arts and Sciences, Newark, Rutgers University*, pp.352-398.

[Ols84] Olsen, P.E. and Galton, P.M. 1984. A review of the reptile and amphibian assemblages from the Stormberg of southern Africa, with special emphasis on the footprints and the age of the Stormberg. *Palaeontologica Africana*, 25, pp.87-110.

[Ols98] Olsen, P.E., Smith, J.B. and McDonald, N.G. 1998. Type material of the type species of the classic theropod footprint genera *Eubrontes*, *Anchisauripus*, and *Grallator* (Early Jurassic, Hartford and Deerfield basins, Connecticut and Massachusetts, USA). *Journal of vertebrate Paleontology*, 18 (3), pp.586-601. doi: 10.1080/02724634.1998.10011086

[Ols03] Olsen, P.E. and Rainforth, E.C. 2003. The early Jurassic ornithischian dinosaurian ichnogenus Anomoepus. *The great rift valleys of Pangea in eastern North America*, 2, pp.314-367.

[Ouk22] Oukassou, M., Zafaty, O., Gierliński, G.D., Klein, H., Saber, H., Amzil, M. and Charrière, A. 2022. First record of a small stegosaur footprint (cf. *Stegopodus*) from the? Upper Jurassic-? Lower Cretaceous red beds of the Middle Atlas, Morocco. *Ichnos*, 29 (3-4), pp.195-204. doi: 10.1080/10420940.2023.2182299

[Pan24] Panascí, G., Varricchio, D.J., Martin, A.J. and Dyman, T. 2024. Dinosaur tracks from the Frontier Formation, Montana: preservation, distribution and palaeoecological significance for the middle Cretaceous terrestrial ecosystems of North America. *Historical Biology*, 36 (4), pp.796-819. doi: 10.1080/08912963.2023.2184692

[Pas09] Pascual-Arribas, C., Hernández Medrano, N., Latorre Macarrón, P. and Sanz Pérez, E. 2009. El icnogénero *Iguanodontipus* en el yacimiento de “Las 17 Cuestas I” (Santa Cruz de Yanguas, Soria, España). *Studia Geologica Salmanticensia*, 45, pp.105-128. doi:

[Pas12] Pascual-Arribas, C., Canudo, J.I., Hernández Medrano, N., Barco, J.L. and Castanera, D. 2012. First record of stegosaur dinosaur tracks in the Lower Cretaceous (Berriasian) of Europe (Oncala group, Soria, Spain). *Geodiversitas*, 34 (2), pp.297-312. doi: 10.5252/g2012n2a4

[Pat04] Patterson, J. and Lockley, M.G. 2004. A probable Diatryma track from the Eocene of Washington: an intriguing case of controversy and skepticism. *Ichnos*, 11 (3-4), pp.341-347. doi: 10.1080/10420940490442278

[Per97] Pérez-Lorente, F., Cuenca-Bescós, G., Aurell, M., Canudo, J.I., Soria, A.R. and Ruiz-Omeñaca, J.I. 1997. Las Cerradicas tracksite (Berriasian, Galve, Spain): growing evidence for quadrupedal ornithopods. *Ichnos*, 5 (2), pp.109-120. doi: 10.1080/10420949709386410

[Per15] Pérez-Lorente, F. 2015. Dinosaur Footprints and Trackways of La Rioja. Bloomington: Indiana University Press.

[Pet20] Petti, F.M., Antonelli, M., Citton, P., Mariotti, N., Petruzzelli, M., Pignatti, J., D'Orazi Porchetti, S., Romano, M., Sacchi, E., Sacco, E. and Wagensommer, A. 2020. Cretaceous tetrapod tracks from Italy: a treasure trove of exceptional biodiversity. In Romano M., Citton P. (eds.), Tetrapod ichnology in Italy: the state of the art. *Journal of Mediterranean Earth Sciences*, 12, pp.167-191. doi: 10.3304/jmes.2020.16873

[Pit89] Pitmann, J.G. 1989. Stratigraphy, lithology, depositional, environment, and track type of dinosaur track-bearing beds of the Gulf Coastal Plain. In Gillette, D.D. and Lockley, M.G. (eds.) Dinosaur Tracks and Traces. Cambridge: Cambridge University Press. 135–153 pp.

[Pon14] Pond, S., Lockley, M.G., Lockwood, J.A., Breithaupt, B.H. and Matthews, N.A. 2014. Tracking dinosaurs on the Isle of Wight: a review of tracks, sites, and current research. *Biological Journal of the Linnean Society*, 113 (3), pp.737-757. doi: 10.1111/bij.12340

[Raa72] Raath, M.A. 1972. First record of dinosaur footprints from Rhodesia. *Arnoldia (Rhodesia) Series of miscellaneous publications National Museums of Rhodesia*, 5, pp.1–5.

[Rai96] Rainforth, E.C. and Lockley, M.G. 1996. Tracking life in a Lower Jurassic desert: vertebrate tracks and other traces from the Navajo Sandstone. In Morales, M. (ed.) The Continental Jurassic. *Museum of Northern Arizona Bulletin*, 60, pp.285-289.

[Rau18] Rauhut, O.W., Pinuela, L., Castanera, D., García-Ramos, J.C. and Cela, I.S. 2018. The largest European theropod dinosaurs: remains of a gigantic megalosaurid and giant theropod tracks from the Kimmeridgian of Asturias, Spain. *PeerJ*, 6, p.e4963. doi: 10.7717/peerj.4963

[Raz16] Razzolini, N.L., Vila, B., Díaz-Martínez, I., Manning, P.L. and Galobart, À. 2016. Pes shape variation in an ornithopod dinosaur trackway (Lower Cretaceous, NW Spain): new evidence of an antalgic gait in the fossil track record. *Cretaceous Research*, 58, pp.125-134. doi: 10.1016/j.cretres.2015.10.012

[Raz17] Razzolini, N.L., Belvedere, M., Marty, D., Paratte, G., Lovis, C., Cattin, M. and Meyer, C.A. 2017. *Megalosauripus transjuranicus* ichnosp. nov. A new Late Jurassic theropod ichnotaxon from NW Switzerland and implications for tridactyl dinosaur ichnology and ichnotaxomy. *Plos one*, 12 (7), p.e0180289. doi: 10.1371/journal.pone.0180289

[Rey89] Reynolds, R.E. 1989. Dinosaur trackways in the Lower Jurassic Aztec Sandstone of California. In Gillette, D.D. and Lockley, G.M. (eds.) Dinosaur Tracks and Traces. Cambridge: Cambridge University Press. 285–292 pp.

[Rod12] Rodríguez-de la Rosa, R.A., Bravo-Cuevas, V.M., Carrillo-Montiel, E. and Ortiz-Ubilla, A., 2012. Lower Cretaceous Dinosaur Tracks from Puebla, Mexico. *Journal of Geological Research*, 2012 (1), p.808729. doi: 10.1155/2012/808729

[Rom21] Romilio, A., Salisbury, S.W. and Jannel, A. 2021. Footprints of large theropod dinosaurs in the Middle–UpperJurassic (lower Callovian–lower Tithonian) Walloon Coal Measures of southern Queensland, Australia. *Historical Biology*, 33 (10), pp.2135-2146. doi: 10.1080/08912963.2020.1772252

[Sal16] Salisbury, S.W., Romilio, A., Herne, M.C., Tucker, R.T. and Nair, J.P. 2016. The dinosaurian ichnofauna of the lower cretaceous (Valanginian–Barremian) broome sandstone of the Walmadany area (James Price Point), Dampier Peninsula, Western Australia. *Journal of Vertebrate Paleontology*, 36 (sup1), pp.1-152. doi: 10.1080/02724634.2016.1269539

[San13] Santos, V.F., Callapez, P.M. and Rodrigues, N.P.C. 2013. Dinosaur footprints from the Lower Cretaceous of the Algarve Basin (Portugal): New data on the ornithopod palaeoecology and palaeobiogeography of the Iberian Peninsula. *Cretaceous Research*, 40, pp.158-169. doi: 10.1016/j.cretres.2012.07.001

[San09] Santos, V.F., Moratalla, J.J. and Royo-Torres, R. 2009. New sauropod trackways from the Middle Jurassic of Portugal. *Acta Palaeontologica Polonica*, 54 (3), pp.409-422. doi: 10.4202/app.2008.0049

[San24] Santos, V.F., Moratalla, J.J., Royo-Torres, R., Belvedere, M., Castanera, D., Ceríaco, L.M., Mocho, P., Merino, M., Meyer, C.A. and Sciscio, L. 2024. A revised name and new insights into the Middle Jurassic sauropod trackways from Portugal. A correction of Santos et al. 2009. *Acta Palaeontologica Polonica*, 69 (4), pp.587-590. doi: 10.4202/app.01167.2024

[Sar74] Sarjeant, W.A.S. 1974. A history and bibliography of the study of fossil vertebrate footprints in the British Isles. *Palaeogeography, Palaeoclimatology, Palaeoecology*, 16 (4), pp.265-378. doi: 10.1016/0031-0182(74)90024-8

[Sar98] Sarjeant, W.A.S., Delair, J.B. and Lockley, M.G. 1998. The footprints of Iguanodon: a history and taxonomic study. *Ichnos: An International Journal of Plant & Animal*, 6 (3), pp.183-202. doi: 10.1080/10420949809386448

[Sar01] Sarjeant, W.A.S. and Reynolds, R.E. 2001, April. Bird footprints from the Miocene of California. In Reynolds, R.E. (ed.) *The changing face of the east Mojave Desert: Abstracts from the 2001 Desert Symposium*. California: California State University. pp.21-40.

[Ser22] Serrano-Brañas, C.I., Espinosa-Chávez, B., Ventura, J.F., Barrera-Guevara, D., Torres-Rodríguez, E. and Vega, F.J. 2022. New insights on the avian trace fossil record from NE Mexico: Evidences on the diversity of latest Maastrichtian web-footed bird tracks. *Journal of South American Earth Sciences*, *113*, p.103686. doi: 10.1016/j.jsames.2021.103686

[Shi19] Shillito, A.P. and Davies, N.S. 2019. Dinosaur-landscape interactions at a diverse early Cretaceous tracksite (Lee Ness sandstone, Ashdown Formation, southern England). *Palaeogeography, Palaeoclimatology, Palaeoecology*, 514, pp.593-612. doi: 10.1016/j.palaeo.2018.11.018

[Sho89] Shounan, Z., Jianjun, L., Chenggang, R., Mateer, N. J. and Lockley, M.G. 1989. A review of dinosaur footprints from China. In Gillette, D.D. and Lockley, M.G. (eds.) Dinosaur Tracks and Traces. Cambridge: Cambridge University Press. 187–197 pp.

[Thu94] Thulborn, R.A. 1994. Ornithopod dinosaur tracks from the Lower Jurassic of Queensland. *Alcheringa*, 18 (3), pp.247-258.

[Tsu18] Tsukiji, Y., Azuma, Y., Shiraishi, F., Shibata, M. and Noda, Y. 2018. New ornithopod footprints from the Lower Cretaceous Kitadani Formation, Fukui, Japan: Ichnotaxonomical implications. *Cretaceous Research*, 84, pp.501-514. doi: 10.1016/j.cretres.2017.12.011

[Vil13] Vila, B., Oms, O., Fondevilla, V., Gaete, R., Galobart, A., Riera, V. and Canudo, J.I. 2013. The latest succession of dinosaur tracksites in Europe: hadrosaur ichnology, track production and palaeoenvironments. *PloS one*, 8 (9), p.e72579. doi: 10.1371/journal.pone.0072579

[Wag12] Wagensommer, A., Latiano, M., Leroux, G., Cassano, G. and D’Orazi Porchetti, S. 2012. New dinosaur tracksites from the Middle Jurassic of Madagascar: ichnotaxonomical, behavioural and palaeoenvironmental implications. *Palaeontology*, 55 (1), pp.109-126. doi: 10.1111/j.1475-4983.2011.01121.x

[Wag16] Wagensommer, A., Latiano, M., Mocke, H.B. and D'Orazi Porchetti, S. 2016. Dinosaur diversity in an Early Jurassic African desert: the significance of the Etjo Sandstone ichnofauna at the Otjihaenamaparero locality (Namibia). *Neues Jahrbuch für Geologie und Paläontologie-Abhandlungen*, 281 (2), pp.155-182. doi: 10.1127/njgpa/2016/0593

[Wag23] Wagensommer, A., Dolch, R., Ratolojanahary, T., Donato, S. and Porchetti, S.D.O., 2023. Defining the Bemaraha megatracksite: an update on dinosaur ichnology in Madagascar. In Cónsole-Gonella, C., de Valais, S., Díaz-Martínez, I., Citton, P., Verde, M. and McIlroy D. (eds.) Ichnology in Shallow-marine and Transitional Environments. *Geological Society, London, Special Publications*, 522, London: Geological Society. pp.265-284. doi: 10.1144/SP522-2021-86

[Wan16] Wang, B., Li, J., Bai, Z., Gao, J., Dong, S., Hu, B., Zhao, S. and Chang, J. 2016. Research on Dinoasur Footprints in Zizhou, Shaanxi Province, China. *Acta Geologica Sinica (English Edition)*, 90 (1), pp.1-18. doi: 10.1111/1755-6724.12639

[Wee06] Weems, R.E. 2006. The manus print of *Kayentapus minor*; its bearing on the biomechanics and ichnotaxonomy of Early Mesozoic saurischian dinosaurs. In Harris, J.D., Lucas, S.G., Spielmann, J.A., Lockley, M.G., Milner, A.R.C. and Kirkland, J.I. (eds.) The Triassic-Jurassic Terrestrial Transition. *New Mexico Museum of Natural History and Science Bulletin*, 37, pp.369–378.

[Wee18] Weems, R.E., 2018. A synopsis of the vertebrate fauna from the Culpeper Basin (Upper Triassic–Lower Jurassic, Maryland and Virginia). In Lucas, S.G. and Sullivan, R.M. Fossil Record 6. *New Mexico Museum of Natural History and Science, Bulletin*, 79, pp.749-768.

[Why94] Whyte, M.A. and Romano, M. 1994. Probable sauropod footprints from the Middle Jurassic of Yorkshire, England. *Gaia*, 10, pp.15-26.

[Wil09] Wilson, J.A., Marsicano, C.A. and Smith, R.M. 2009. Dynamic locomotor capabilities revealed by early dinosaur trackmakers from Southern Africa. *PLoS One*, 4 (10), p.e7331. doi: 10.1371/journal.pone.0007331

[Woo89] Woodhams, K.E. and Hines, J.S. 1989. Dinosaur footprints from the Lower Cretaceous of East Sussex, England. In Gillette, D.D. and Lockley, G.M. (eds.) Dinosaur Tracks and Traces. Cambridge: Cambridge University Press. 301–307 pp.

[Wri98] Wright, J.L., Barrett, P.M., Lockley, M.G. and Cook, E., 1998. A review of the Early Cretaceous terrestrial vertebrate track-bearing strata of England and Spain. In Lucas, S.G., Kirkland, J.I. and Estep, J.W. Lower and Middle Cretaceous Terrestrial Ecosystems. *New Mexico Museum of Natural History and Science Bulletin*, 14, pp.143-154.

[Xin11] Xing, L., Harris, J.D., Jia, C.K., Luo, Z.J., Wang, S.N. and An, J.F. 2011. Early Cretaceous bird-dominated and dinosaur footprint assemblages from the northwestern margin of the Junggar Basin, Xinjiang, China. *Palaeoworld*, 20 (4), pp.308-321. doi: 10.1016/j.palwor.2011.01.001

[Xin13a] Xing, L., Lockley, M.G., Chen, W., Gierliński, G.D., Li, J., Persons IV, W.S., Matsukawa, M., Ye, Y., Gingras, M.K. and Wang, C. 2013a. Two theropod track assemblages from the Jurassic of Chongqing, China, and the Jurassic stratigraphy of Sichuan Basin. *Vertebrata PalAsiatica*, 51 (2), pp.107-130.

[Xin13b] Xing, L., Klein, H., Lockley, M.G. and Chen, W. 2013b. Earliest records of theropod and mammal-like tetrapod footprints in the Upper Triassic of Sichuan Basin, China. *Vertebrata Palasiatica*, 51 (3), pp.184-198.

[Xin13c] Xing, L., Liu, Y.Q., Kuang, H.W., Klein, H., Zhang, J.P., Burns, M.E., Chen, J., Wang, M.W. and Hu, J. 2013c. Theropod and possible ornithopod track assemblages from the Jurassic–Cretaceous boundary Houcheng Formation, Shangyi, northern Hebei, China. *Palaeoworld*, *23*(2), pp.200-208. doi: 10.1016/j.palwor.2013.10.002

[Xin13d] Xing, L., Lockley, M.G., Li, Z.D., Klein, H., Zhang, J.P., Gierliński, G.D., Ye, Y., Persons IV, W.S. and Zhou, L. 2013d. Middle Jurassic theropod trackways from the Panxi region, Southwest China and a consideration of their geologic age. *Palaeoworld*, 22 (1-2), pp.36-41. doi: 10.1016/j.palwor.2012.11.002

[Xin13e] Xing, L., Lockley, M.G., McCrea, R.T., Gierliński, G.D., Buckley, L.G., Zhang, J., Qi, L. and Jia, C. 2013e. First record of Deltapodus tracks from the Early Cretaceous of China. *Cretaceous Research*, 42, pp.55-65. doi: 10.1016/j.cretres.2013.01.006

[Xin13f] Xing, L., Lockley, M.G., Klein, H., Zhang, J.P., He, Q., Divay, J.D., Qi, L.Q. and Jia, C.K. 2013f. Dinosaur, bird and pterosaur footprints from the Lower Cretaceous of Wuerhe asphaltite area, Xinjiang, China, with notes on overlapping track relationships. *Palaeoworld*, 22 (1-2), pp.42-51. doi: 10.1016/j.palwor.2013.03.001

[Xin14a] Xing, L., Peng, G.Z., Ye, Y., Lockley, M.G., McCrea, R.T., Currie, P.J., Zhang, J.P. and Burns, M.E. 2014a. Large theropod trackway from the Lower Jurassic Zhenzhuchong Formation of Weiyuan County, Sichuan Province, China: review, new observations and special preservation. *Palaeoworld*, 23 (3-4), pp.285-293. doi: 10.1016/j.palwor.2014.10.010

[Xin14b] Xing, L., Lockley, M.G., Zhang, J., Klein, H., Persons IV, W.S. and Dai, H. 2014b. Diverse sauropod-, theropod-, and ornithopod-track assemblages and a new ichnotaxon *Siamopodus* xui ichnosp. nov. from the Feitianshan Formation, Lower Cretaceous of Sichuan Province, southwest China. *Palaeogeography, Palaeoclimatology, Palaeoecology*, *414*, pp.79-97. doi: 10.1016/j.palaeo.2014.08.011

[Xin14c] Xing, L., Klein, H., Lockley, M. G., Wetzel, A., Li, Z., LI, J., Gierliński, G.D., Zhang, J., Matsukawa, M., Divay, J. D. and Zhou, L. 2014c. Changpeipus (theropod) tracks from the Middle Jurassic of the Turpan Basin, Xinjiang, Northwest China: review, new discoveries, ichnotaxonomy, preservation and paleoecology. *Vertebrata PalAsiatica*, 4, 233–259.

[Xin14d] Xing, L., Lockley, M.G., Klein, H., Gierliński, G.D., Divay, J.D., Hu, S.M., Zhang, J.P., Ye, Y. and He, Y.P. 2014d. The non-avian theropod track Jialingpus from the Cretaceous of the Ordos Basin, China, with a revision of the type material: implications for ichnotaxonomy and trackmaker morphology. *Palaeoworld*, 23 (2), pp.187-199. doi: 10.1016/j.palwor.2013.12.001

[Xin14e] Xing, L., Peng, G., Ye, Y., Lockley, M.G., Klein, H., Persons IV, W.S., Zhang, J., Shu, C. and Hao, B. 2014e. Sauropod and small theropod tracks from the Lower Jurassic Ziliujing Formation of Zigong City, Sichuan, China, with an overview of Triassic–Jurassic dinosaur fossils and footprints of the Sichuan Basin. *Ichnos*, 21 (2), pp.119-130. doi: 10.1080/10420940.2014.909352

[Xin14f] Xing, L., Lockley, M.G., Zhang, J., Klein, H., Kim, J.Y., Persons IV, W.S., Matsukawa, M., Yu, X., Li, J., Chen, G. and Hu, Y. 2014f. Upper Cretaceous dinosaur track assemblages and a new theropod ichnotaxon from Anhui Province, eastern China. *Cretaceous Research*, 49, pp.190-204. doi: 10.1016/j.cretres.2014.03.003

[Xin14g] Xing, L., Li, D., Lockley, M. G., Zhang, J.P., You, H., Klein, H., Marty, D., Persons IV, W. S. and Peng, C. 2014g. Theropod and sauropod track assemblages from the Lower Cretaceous Hekou group of Zhongpu, Gansu Province, China. *Acta Palaeontologica Sinica*, 53 (3), pp.381–391.

[Xin14h] Xing, L., Lockley, M.G., Wang, Q.F., Li, Z.D., Klein, H., Persons IV, W.S., Ye, Y. and Matsukawa, M. 2014h. Earliest records of dinosaur footprints in Xinjiang, China. *Vertebrata PalAsiatica*, 52 (3), pp.340-348.

[Xin14i] Xing, L. and Lockley, M.G., 2014i. First report of small Ornithopodichnus trackways from the Lower Cretaceous of Sichuan, China. *Ichnos*, 21 (4), pp.213-222. doi: 10.1080/10420940.2014.951998

[Xin14j] Xing, L., Lockley, M.G., Miyashita, T., Klein, H., Wang, T., Persons IV, W.S., Pan, S.G., Zhang, J.P. and Dong, Z.M. 2014j. Large sauropod and theropod tracks from the Middle Jurassic Chuanjie Formation of Lufeng County, Yunnan Province and palaeobiogeography of the Middle Jurassic sauropod tracks from southwestern China. *Palaeoworld*, 23 (3-4), pp.294-303. doi: 10.1016/j.palwor.2014.04.003

[Xin14k] Xing, L., Niedźwiedzki, G., Lockley, M.G., Zhang, J.P., Cai, X.F., Persons IV, W.S. and Ye, Y. 2014k. Asianopodus-type footprints from the Hekou Group of Honggu District, Lanzhou City, Gansu, China and the "heel" of large theropod tracks. *Palaeoworld*, 23 (3-4), pp.304-313. doi: 10.1016/j.palwor.2014.08.003

[Xin14l] Xing, L., Belvedere, M., Buckley, L., Falk, A.R., Lockley, M.G., Klein, H., Abbassi, N., Zhang, X. and Tang, Y. 2014l. First record of bird tracks from Paleogene of China (Guangdong Province). *Palaeogeography, Palaeoclimatology, Palaeoecology*, 414, pp.415-425. doi: 10.1016/j.palaeo.2014.08.031

[Xin15a] Xing, L., Peng, G., Lockley, M.G., Ye, Y., Klein, H., McCrea, R.T., Zhang, J. and Persons IV, W.S. 2015a. Saurischian (theropod–sauropod) track assemblages from the Jiaguan Formation in the Sichuan Basin, Southwest China: ichnology and indications to differential track preservation. *Historical Biology*, 28 (8), pp.1003-1013. doi: 10.1080/08912963.2015.1088845

[Xin15b] Xing, L., Lockley, M.G., Marty, D., Zhang, J., Wang, Y., Klein, H., McCrea, R.T., Buckley, L.G., Belvedere, M., Mateus, O. and Gierliński, G.D. 2015b. An ornithopod-dominated tracksite from the Lower Cretaceous Jiaguan Formation (Barremian–Albian) of Qijiang, South-Central China: new discoveries, ichnotaxonomy, preservation and palaeoecology. *PLoS One*, 10 (10), p.e0141059. doi: 10.1371/journal.pone.0141059

[Xin15c] Xing, L., Peng, G.Z., Lockley, M.G., Ye, Y., Klein, H., Zhang, J.P. and Persons IV, W.S., 2015c. Early Cretaceous sauropod and ornithopod trackways from a stream course in Sichuan Basin, Southwest China. In Sullivan, R.M. and Lucas, S.G. (eds.) Fossil Record 4. *New Mexico Museum of Natural History and Science Bulletin*, 68, pp.319-325.

[Xin15d] Xing, L., Lockley, M.G., Tang, Y., Klein, H., Zhang, J., Persons IV, W.S., Dai, H. and Ye, Y. 2015d. Theropod and ornithischian footprints from the middle jurassic yanan formation of zizhou county, shaanxi, China. *Ichnos*, 22 (1), pp.1-11. doi: 10.1080/10420940.2014.985670

[Xin15e] Xing, L., Lockley, M.G., Zhang, J., Klein, H., Marty, D., Peng, G., Ye, Y., McCrea, R.T., Persons IV, W.S. and Xu, T., 2015e. The longest theropod trackway from East Asia, and a diverse sauropod-, theropod-, and ornithopod-track assemblage from the Lower Cretaceous Jiaguan Formation, southwest China. *Cretaceous Research*, *56*, pp.345-362. doi: 10.1016/j.cretres.2015.05.008

[Xin15f] Xing, L., Buckley, L.G., McCrea, R.T., Lockley, M.G., Zhang, J., Piñuela, L., Klein, H. and Wang, F. 2015f. Reanalysis of Wupus agilis (Early Cretaceous) of Chongqing, China as a large avian trace: differentiating between large bird and small non-avian theropod tracks. *PLoS One*, 10 (5), p.e0124039. doi: 10.1371/journal.pone.0124039

[Xin16a] Xing, L., Lockley, M.G., Marty, D., Klein, H., Yang, G., Zhang, J., Peng, G., Ye, Y., Persons IV, W.S., Yin, X. and Xu, T. 2016a. A diverse saurischian (theropod–sauropod) dominated footprint assemblage from the Lower Cretaceous Jiaguan Formation in the Sichuan Basin, southwestern China: a new ornithischian ichnotaxon, pterosaur tracks and an unusual sauropod walking pattern. *Cretaceous Research*, 60, pp.176-193. doi: 10.1016/j.cretres.2015.12.005

[Xin16b] Xing, L., Lockley, M.G., Klein, H., Zhang, J. and Persons IV, W.S. 2016b. A new ornithischian-dominated and theropod footprint assemblage from the Lower Jurassic Lufeng Formation of Yunnan province, China. In Sullivan, R.M. and Lucas, S.G. (eds.) Fossil Record 5. *New Mexico Museum of Natural History and Science Bulletin*, 74, pp.331-338.

[Xin16c] Xing, L., Lockley, M.G., Hu, S., Li., Q. and Persons I.V., W.S. 2016c. Early Jurassic *Anomoepus* track from the Fengjiahe formation of northern central Yunnan, China. In Sullivan, R.M. and Lucas, S.G. (eds.) Fossil Record 5. *New Mexico Museum of Natural History and Science Bulletin*, 74, pp.327-330.

[Xin16d] Xing, L., Abbassi, N., Lockley, M.G., Klein, H., Jia, S., McCrea, R.T. and Persons IV, W.S., 2016d. The first record of *Anomoepus* tracks from the Middle Jurassic of Henan province, Central China. *Historical Biology*, 29 (2), pp.223-229. doi: 10.1080/08912963.2016.1149480

[Xin16e] Xing, L., Lockley, M.G., Wang, Y., Pole, M.S., Klein, H., Peng, G., Xie, X., Zhang, G., Deng, C. and Burns, M.E. 2016e. New middle Jurassic dinosaur track record from northeastern Sichuan Province, China. *Swiss Journal of Palaeontology*, 136 (2), pp.359-364. doi: 10.1007/s13358-016-0123-4

[Xin16f] Xing, L., Lockley, M.G., Klein, H., Falkingham, P.L., Kim, J.Y., Mccrea, R.T., Zhang, J., Persons IV, W.S., Wang, T. and Wang, Z. 2016f. First early Jurassic small ornithischian tracks from Yunnan Province, southwestern China. *Palaios*, 31 (11), pp.516-524. doi: 10.2110/palo.2015.074

[Xin16g] Xing, L., Lockley, M.G., Yang, G., Cao, J., Benton, M., Xu, X., Zhang, J., Klein, H., Persons IV, W.S., Kim, J.Y. and Peng, G. 2016g. A new Minisauripus site from the Lower Cretaceous of China: Tracks of small adults or juveniles?. *Palaeogeography, Palaeoclimatology, Palaeoecology*, 452, pp.28-39. doi: 10.1016/j.palaeo.2016.04.006

[Xin16h] Xing, L., Lockley, M.G., Peng, G.Z., Ye, Y., Zhang, J.P., Matsukawa, M., Klein, H., McCrea, R.T. and Persons IV, W.S. 2016h. Eubrontes and Anomoepus track assemblages from the Middle Jurassic Xiashaximiao Formation of Zizhong County, Sichuan, China: Review, ichnotaxonomy and notes on preserved tail traces. . In Sullivan, R.M. and Lucas, S.G. (eds.) Fossil Record 5. *New Mexico Museum of Natural History and Science Bulletin*, 74, pp.345-352.

[Xin16i] Xing, L., Buckley, L.G., Lockley, M.G., Zhang, J., Marty, D., Wang, Y., Li, J., McCrea, R.T. and Peng, C. 2016i. A new bird track, *Koreanaornis lii* ichnosp. nov., from the Lower Cretaceous Hekou Group in the Lanzhou-Minhe Basin, Gansu, Northwest China, and implications for Early Cretaceous avian diversity. *Cretaceous Research*, 66, pp.141-154. doi: 10.1016/j.cretres.2016.06.005

[Xin17a] Xing, L., Lockley, M.G., Li, D., Klein, H., Ye, Y., Persons IV, W.S. and Ran, H. 2017a. Late Cretaceous ornithopod-dominated, theropod, and pterosaur track assemblages from the Nanxiong Basin, China: New discoveries, ichnotaxonomy, and paleoecology. *Palaeogeography, Palaeoclimatology, Palaeoecology*, 466, pp.303-313. doi: 10.1016/j.palaeo.2016.11.035

[Xin17b] Xing, L.D., Lockley, M.G., Zhang, J.P., Klein, H., Kümmell, S.B., Persons IV, W.S. and Kuang, H.W. 2017b. Theropod tracks from the Lower Jurassic of Gulin area, Sichuan province, China. *Palaeoworld*, 26 (1), pp.115-123. doi: 10.1016/j.palwor.2015.11.003

[Xin18] Xing, L., Buckley, L.G., Lockley, M.G., McCrea, R.T. and Tang, Y. 2018. Lower Cretaceous avian tracks from Jiangsu Province, China: a first Chinese report for ichnogenus *Goseongornipes* (Ignotornidae). *Cretaceous Research*, 84, pp.571-577. doi: 10.1016/j.cretres.2017.12.016

[Xin19a] Xing, L., Niu, K., Lockley, M.G., Klein, H., Romilio, A., Persons IV, W.S. and Brusatte, S.L. 2019a. A probable tyrannosaurid track from the Upper Cretaceous of southern China. *Science Bulletin*, 64, pp.1136–1139. doi: 10.1016/j.scib.2019.06.013

[Xin19b] Xing, L., Lockley, M.G., Klein, H., Gierliński, G.D., Ye, Y., Zhang, J., Persons IV, W.S. and Wang, T., 2019b. First thyreophoran type tracks from the Middle Jurassic Chuanjie Formation of Yunnan Province, China. *Ichnos*, 26 (1), pp.8-15. doi: 10.1080/10420940.2017.1366904

[Xin21a] Xing, L., Lockley, M.G., Klein, H., Zhang, L.J., Romilio, A., Scott Persons IV, W., Peng, G.Z., Ye, Y. and Wang, M.Y. 2021a. The new ichnotaxon Eubrontes nobitai ichnosp. nov. and other saurischian tracks from the Lower Cretaceous of Sichuan Province and a review of Chinese Eubrontes-type tracks. *Journal of Palaeogeography*, *10*(1), p.17.

[Xin21b] Xing, L., Lockley, M.G., Klein, H., Persons IV, W.S., Peng, G., Ye, Y. and Wang, M. 2021. Largest dinosaur tracksite in China (Cretaceous, Zhaojue area, Sichuan Province): on the verge of destruction. *Geoscience frontiers*, *12*(5), p.101181. doi: 10.1016/j.gsf.2021.101181

[Xin21c] Xing, L., Lockley, M.G., Romilio, A., Klein, H., Peng, G., Persons IV, W.S., Ye, Y., Jiang, S. and Wang, M. 2021c. An historic theropod-dominated track assemblage from the Upper Jurassic of Sichuan, China. *Historical Biology*, 33 (11), pp.2822-2828. doi: 10.1080/08912963.2020.1830278

[Xin21d] Xing, L., Lockley, M.G., Jia, C., Klein, H., Niu, K., Zhang, L., Qi, L., Chou, C., Romilio, A., Wang, D. and Zhang, Y. 2021d. Lower cretaceous avian-dominated, theropod, thyreophoran, pterosaur and turtle track assemblages from the Tugulu Group, Xinjiang, China: ichnotaxonomy and palaeoecology. *PeerJ*, 9, p.e11476. doi: 10.7717/peerj.11476

[Xin21e] Xing, L., Lockley, M.G., Peng, G., Ye, Y., Jiang, S., Romilio, A., Persons IV, W.S. and Wang, M. 2021e. A review of two Middle Jurassic theropod tracksites discovered in the 1980s from Sichuan Basin. *Biosis: Biological Systems*, 2 (1), pp.191-208. doi: 10.37819/biosis.002.01.0094

[Xin21f] Xing, L., Peng, C., Lockley, M.G., Wang, Y., Li, D., Klein, H., Yang, J., Li, L., Persons, W.S. and Wang, M., 2021f. A diversified tetrapod ichnite fauna from the Lower Cretaceous Hekou Group of Gansu Province, China. *Historical Biology*, 33 (11), pp.3018-3030.

[Xin21g] Xing, L., Lockley, M.G., Wang, D., Liu, C., Ma, D., Jia, Z., Wei, J. and Persons, W.S. 2021g. Ornithischian tracks from the Middle Jurassic of the Junggar Basin, Xinjiang, Northwest China. *Historical Biology*, 34 (9), pp.1772-1779.

[Xin25a] Xing, L., Niu, K., Chen, Q., Klein, H., Romilio, A., Chen, R., Lin, M., Deng, K. and Tang, J. 2025a. Dinosaur track assemblages from mid-Cretaceous of Fujian Province, southeastern China: ichnotaxonomic review and faunal comparison. *PeerJ*, 13, p.e19597. doi: 10.7717/peerj.19597

[Xin25b] Xing, L., Li, D., Chen, Q., Fu, J., Chou, C., Zhang, L., You, W., Shi, K. and Klein, H. 2025b. Early Cretaceous dinosaur, bird and turtle tracks from the Lanzhou-Minhe Basin, Gansu Province, Northwest China. *Cretaceous Research*, 166, p.105987. doi: 10.1016/j.cretres.2024.105987

[Yoo21] Yoon, H.S., Lee, Y.N., Jung, S.H., Kong, D.Y., Kim, S.H. and Son, M. 2021. A juvenile ornithopod tracksite from the Lower Cretaceous Haman Formation, South Korea. *Cretaceous Research*, 125, p.104877. doi: 10.1016/j.cretres.2021.104877

[Zhe86] Zhen, S., Li, J. and Rao, C. 1986. Dinosaur footprints of Jinning, Yunnan. *Beijing Natural History Museum*, 33, pp.1–19.
